# Increased pine root exudation of phenolic acids and amino acids under drought

**DOI:** 10.1093/treephys/tpag116

**Published:** 2026-08-20

**Authors:** Sophie Obersteiner, Yaara Oppenheimer-Shaanan, Tamir Klein

**Affiliations:** Plant & Environmental Sciences Department, Weizmann Institute of Science, 234 Herzl Street, Rehovot 7610001, Israel; Plant & Environmental Sciences Department, Weizmann Institute of Science, 234 Herzl Street, Rehovot 7610001, Israel; Department of Life Sciences, Achva Academic College, Mobile Post Shikmim, Arugot 7980400, Israel; Plant & Environmental Sciences Department, Weizmann Institute of Science, 234 Herzl Street, Rehovot 7610001, Israel

**Keywords:** aridity, CO_2_ enrichment, carbon allocation, exudate metabolites, non-structural carbohydrates, root secretions

## Abstract

Belowground carbon (C) allocation by trees, particularly through root exudation, is a key pathway influencing long-term C storage in forest soils. Future climate change scenarios, such as drought and elevated CO_2_ (eCO_2_) strongly affect trees’ physiology and influence belowground C allocation. However, how the rate and chemical composition of root exudation change, especially under the combined effect of drought and eCO_2_, remains poorly understood. We conducted an experiment to examine the single and combined effects of drought and eCO_2_ on the belowground C allocation of *Pinus brutia* Ten saplings. Root exudates were collected, quantified and metabolically profiled. Our results revealed that under eCO_2_, C assimilation increased up to 2.2-fold, increasing plant biomass, while the root exudation rate and composition remained unchanged under eCO_2_ compared with ambient CO_2_ (aCO_2_). Additionally, when trees were exposed to drought stress, the root exudation rate increased 4.3-fold under aCO_2_ and up to 10.4-fold when combined with eCO_2_; however, overall, eCO_2_ had no effect on root exudation. Root starch reserves decreased under drought, whereas soluble sugars increased, with the largest increase under combined drought and eCO_2_. Further, drought altered exudate composition, increasing several metabolites, mainly phenolic acids (e.g., gallic acid and caffeic acid), amino acids and key osmoprotectants such as trehalose, proline and betaine. Overall, these observations show that pines strongly increased their root exudation under drought and that exudate metabolites shifted towards more specialized metabolites linked to stress metabolism.

## Introduction

Root exudation is a major pathway of belowground carbon (C) allocation in trees, transferring recently fixed photosynthates to the rhizosphere (Chari et al. 2024, Rog et al. 2024). These exudates comprise primary metabolites such as sugars, amino acids and organic acids, as well as secondary, more specialized metabolites, all of which influence nutrient acquisition, microbial activity and soil C cycling (Oburger and Jones 2018, Canarini et al. 2019, Oppenheimer-Shaanan et al. 2022, Panchal et al. 2022). Their rate and composition vary depending on plant species, developmental stage, root traits, nutrient availability, soil type, and abiotic and biotic conditions (Badri and Vivanco 2009, Meier et al. 2013, Baetz and Martinoia 2014, Korenblum et al. 2020, Robert et al. 2025). Among abiotic drivers, intensified drought and elevated CO₂ (eCO₂) can alter both the quantity and chemistry of root exudates (Phillips et al. 2011, Sánchez-Carrillo et al. 2018, Brunn et al. 2025). Their interaction may produce additive or non-additive effects on root exudation, potentially altering its ecological role in the C cycle (Zandalinas and Mittler 2022, Umaña et al. 2025).

In trees, root exudation can represent a substantial C sink and should therefore be considered within the whole-tree C budget. Trees can assimilate C for growth, store C as starch, allocate C as soluble sugars, release C via root exudation and deposit C as litter (Klein and Hoch 2015, Sell et al. 2024). A mechanistic understanding of C allocation therefore requires addressing three linked questions: to which pools C is allocated, how much C is allocated to each pool and in what chemical form C is allocated. Drought reduces stomatal conductance to limit hydraulic failure but restricts assimilation (McDowell et al. 2008, Lin et al. 2015). By contrast, CO_2_ fertilization can increase assimilation and improve water-use efficiency (WUE), potentially mitigating drought effects (Wullschleger et al. 2002, Avila et al. 2020, Birami et al. 2020, Dror and Klein 2021). While drought and eCO_2_ effects on growth and non-structural carbohydrates (NSCs) have been widely studied, only in recent years have a growing number of studies quantified root exudation responses to drought and eCO_2_ in trees (Dror and Klein 2021, Yin et al. 2023, W.Li et al. 2025, Reay et al. 2025). Drought often increases tree root exudation, although decreases have also been reported, likely reflecting variation in drought intensity, duration and species-specific C allocation strategies (Brunn et al. 2025). Under eCO_2_, increased assimilation can enhance belowground allocation, particularly when growth is nutrient limited (Phillips et al. 2011, Jiang et al. 2020, da Silva Fortirer et al. 2023, Zeng et al. 2024, Reay et al. 2025, Umaña et al. 2025). Under combined eCO_2_ and drought, biomass responses are often largely additive (Umaña et al. 2025), but whether root exudation follows similar patterns remains unclear. Improved WUE under eCO_2_ could reduce drought-driven exudation, although current evidence is limited and inconsistent (Calvo et al. 2017, Zeng et al. 2024). Moreover, most studies focus on exudation rates, whereas chemically resolved responses remain scarce, especially under combined drought and eCO_2_. Thus, how drought and eCO₂ interact to regulate both the rate and chemistry of tree root exudation remains poorly understood.

Many studies describe root exudation as an active C investment, whereby plants adjust the exudation rate and composition to mobilize nutrients, recruit beneficial microbes, hence potentially enhancing stress tolerance (Canarini et al. 2019, Oppenheimer-Shaanan et al. 2022, J.Li et al. 2025). Alternatively, surplus-C theory frames exudation as an outcome of source–sink imbalance, where excess assimilates are redirected belowground when growth and storage sinks are constrained (Prescott et al. 2020, Prescott 2022). At drought onset, growth may decline before assimilation does, creating a transient surplus-C state that could increase soluble sugars and fuel exudation (Prescott et al. 2020). Root NSC has been reported to correlate with exudation rates (Yin et al. 2023, W.Li et al. 2025), although such relationships are not universal (Karst et al. 2017). Exudation quantity and chemistry may therefore provide complementary insight: the amount of C released may reflect source–sink balance, whereas chemical composition may indicate a more selective response.

Recent advances in metabolomics now enable broader characterization of the chemical composition of tree root exudates and the detection of previously unreported compounds (Ritter et al. 2025). Root exudates comprise a diverse mixture of primary metabolites, which are involved in microbial turnover and nutrient mobilization, and secondary metabolites, which can mediate defence, allelopathy and plant–microbe signaling (Badri and Vivanco 2009, Zhalnina et al. 2018, Canarini et al. 2019, Bhatla and Lal 2023). These compound classes may also differ in their regulation: primary metabolites are often more closely linked to concentration gradients and facilitated diffusion, whereas many secondary metabolites are released through more selective, transporter-mediated pathways (Canarini et al. 2019). Exudate chemistry may therefore provide additional insight into the processes underlying changes in the exudation rate. Drought may promote the production of stress-related secondary metabolites, particularly phenolics (Yadav et al. 2021), with tree studies reporting shifts in root exudates toward phenolics and terpenoids (Gargallo-Garriga et al. 2018, Oppenheimer-Shaanan et al. 2022). Broader plant meta-analyses indicate increases in some carboxylates under drought and in carbohydrates and organic acids under eCO_2_ (Zeng et al. 2024). Because exudate composition can overlap strongly with root metabolism (Liu et al. 2025), metabolite profiles may provide insight into both rhizosphere functioning and internal plant stress responses.

Here, we examined the effects of single and combined exposure to drought and eCO_2_ on C allocation in *Pinus brutia* Ten, focusing on root exudation rate and composition. *Pinus brutia* is a dominant native conifer in eastern Mediterranean forests and is widely used for afforestation in water-limited regions (Chambel et al. 2013), making it a relevant model for climate-change responses in Mediterranean ecosystems. Using custom-made rhizoboxes that permit repeated access to intact roots, we aimed to (i) examine root exudation rates in relation to other C pools, including assimilation, growth, storage and soluble sugars, across CO_2_ levels (aCO_2_ and eCO_2_) and drought phases (pre-drought, drought and post-drought recovery) and (ii) assess changes in root exudate metabolite groups across these phases. We hypothesized that (i) under eCO_2_, increased C assimilation would primarily support growth, but eventually, growth might be constrained; hence, root exudation could increase; (ii) under drought, reduced growth would increase belowground C availability and exudation, accompanied by a shift toward stress-associated metabolites, particularly phenolics and terpenoids; and (iii) under combined eCO_2_ × drought, improved WUE under eCO_2_ would alleviate drought-driven growth limitation and thereby reduce the drought-induced exudation response relative to aCO_2_ × drought.

## Materials and methods

### Plant and soil material

*Pinus brutia* seedlings (~7 months old) were obtained from the nursery of the Jewish National Fund (JNF, the Israeli forestry agency, Eshtaol, Israel). The seedlings were potted in custom-made 25 L containers termed ‘Rhizoboxes’ for ~9 months to enable the root system to develop, in a shaded net-house at the Weizmann Institute, Rehovot, Israel. The Rhizoboxes utilized in the study are transparent plastic containers equipped with doors on each side, allowing convenient access to the root system and soil (Oppenheimer-Shaanan et al. 2022; Figure S1A available as Supplementary Data at *Tree Physiology* Online). To enhance the ecological relevance of the study and to maintain the presence of naturally occurring soil microbiota, the soil was collected from a Mediterranean mixed forest, located at the Judean foothills (31° 43*′*N 34° 57*′*E, 320 m elevation), Israel. The collected forest soil, classified as silty clay loam, had high silt (48%) and clay (33%) content, which can limit proper drainage, aeration and root growth due to its high bulk density. To improve these properties, we mixed the soil with washed siliceous sand in a 1:1 ratio and added 0.5 L of tuff (a porous volcanic rock) per 10 L of the sand–soil mixture. This ratio was chosen to achieve a soil texture classified as sandy loam (59% sand, 24% silt and 17% clay), known for its optimal water retention, aeration and root zone oxygen diffusion, which supports better physicochemical conditions for plant growth (Pragg et al. 2024, Xu et al. 2024). Soil texture analysis was performed using PARIO (Meter, Munich, Germany), following the manufacturer’s protocol.

### Experimental design and treatments

**Figure 1:**
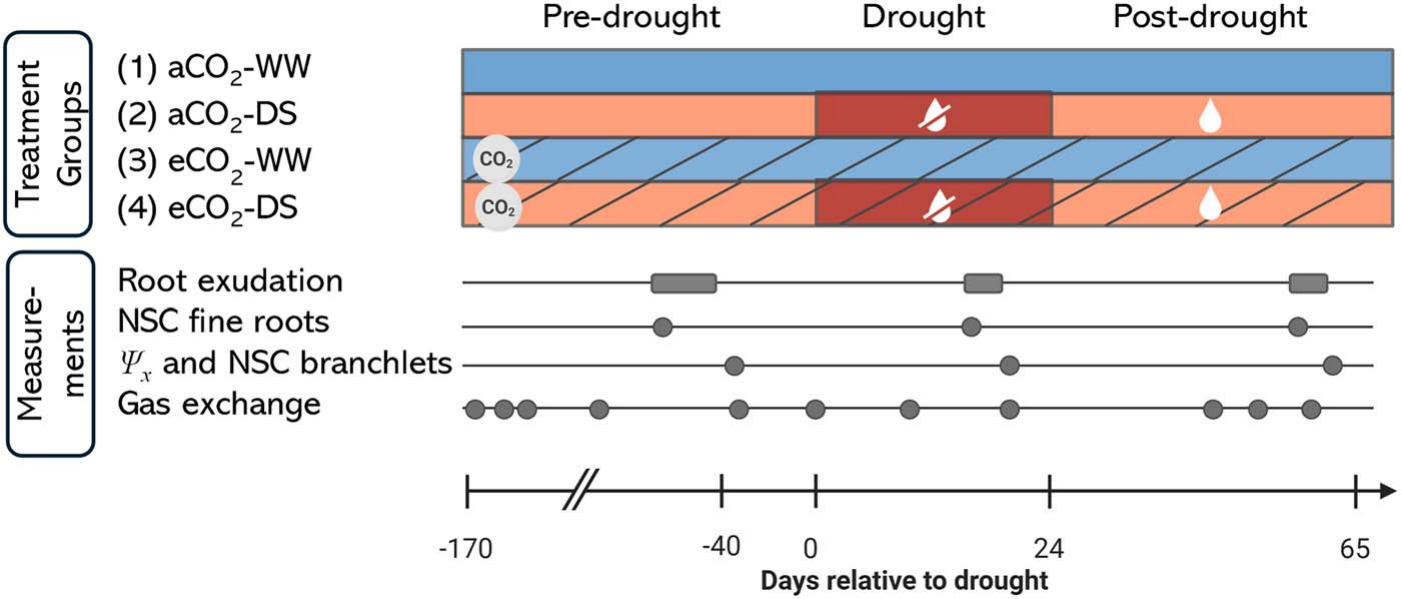
Experimental setup: pines growing in Rhizoboxes in growth rooms were divided into four groups (*n* = 8): (1) aCO_2_-WW, (2) aCO_2_-DS, (3) eCO_2_-WW and (4) eCO_2_-DS with 24 days of drought followed by 42 days of re-irrigation; where aCO_2_ = 400 p.p.m., eCO_2_ = 850 p.p.m., WW = well-watered, DS = drought-stressed; NSC = non-structural carbohydrate; *Ψ*_x_ = stem water potential. Created in https://BioRender.com.

The 32 tree saplings (~ 16 months old) were randomly distributed to two identical climate-controlled growth rooms (Dror and Klein 2021). Saplings were divided into four treatment groups (*n* = 8) in a 2 × 2 factorial design with two CO_2_ levels (aCO_2_ = ambient, eCO_2_ = elevated) and two drought levels (WW = well-watered, DS = drought-stressed and re-irrigation). The treatment groups were: (1) aCO_2_-WW, (2) aCO_2_-DS, (3) eCO_2_-WW and (4) eCO_2_-DS (Figure 1). Measurements were conducted at three time-periods relative to drought onset (days relative to drought (DRD) = 0): pre-drought (after CO_2_ acclimation; DRD −170 to 0), drought (DRD 0–24) and post-drought recovery (after re-irrigation; DRD 24–65; Figure 1). Planned biological replication was *n* = 8 per treatment and time point for all measurements, except exudate metabolite analysis (*n* = 5; see final replicate numbers in Table S1 available as Supplementary Data at *Tree Physiology* Online). Repeated measurements were made on the same trees across all three time points.

After 2 months of room-acclimation of sapling (DRD −191), the CO_2_ level of one room was set to 850 p.p.m. (eCO_2_), and the other remained at ambient CO_2_ (aCO_2_ ~400 p.p.m.). Days of exposure of saplings to eCO_2_ were increased gradually in the first month of CO_2_ acclimation. To minimize any potential room effect, saplings and CO_2_ levels were switched monthly, except for the third month of CO_2_ acclimation, due to technical challenges. eCO_2_ was regulated by a system comprising a compressed CO_2_ cylinder and an infrared CO_2_ sensor (0–2000 p.p.m. CO_2_, PolyGard Transmitter, Pocking, Germany), maintaining CO_2_ concentration around 850 p.p.m. Short-term fluctuations occurred due to routine handling including room changes, human presence and replacement of gas cylinders. CO_2_ concentration was logged using an SCD30 sensor with a measurement accuracy of ±30 p.p.m. at a range of 400–10,000 p.p.m. (Sensirion, Chicago, IL, USA) connected to an ESP32 controller (Espressif Systems, Shanghai, China; Figure S1B available as Supplementary Data at *Tree Physiology* Online). Day and night temperatures were set to 26 °C in both rooms. Relative humidity was between 50% and 75%. The light level was controlled by an array of high-intensity LED lamps (Regiolux, Königsberg, Germany and Nisko, Modi’in, Israel), producing a photosynthetically active radiation that ranged from ~200 μmol photons m^−2^ s^−1^ at pot height to up to ~900 μmol photons m^−2^ s^−1^ at average treetop.

From DRD −41 onward, the saplings received 0.6 L of water daily. After 6 months of CO_2_ acclimation (DRD 0), half of the trees (eight trees in each room) were randomly subjected to drought conditions. Drought was induced by withholding irrigation for 24 days, with the aim of reaching the species-specific water potential value of 50% loss of hydraulic conductivity (P50) of pines (between −4.0 and −4.5 MPa of stem water potential), ensuring that the trees experienced severe drought stress (Wagner et al. 2023). This goal was achieved with drought-stressed trees reaching −4.4 ± 0.16 MPa of stem water potential (Figure S2 available as Supplementary Data at *Tree Physiology* Online). Subsequently, drought-stressed saplings were re-irrigated for an additional 42 days, during which stem water potential of drought-stressed trees recovered to −1.06 ± 0.09 MPa, nearly matching the well-watered trees (−0.95 ± 0.07 MPa; Figure S2 available as Supplementary Data at *Tree Physiology* Online), confirming that the treatment imposed a severe but non-lethal drought. During the experiment the saplings received no fertilization.

Because belowground measurements are highly sensitive to disturbance and oxygen exposure, each exudation campaign used a new, previously unopened Rhizobox window (out of a total of four), ensuring that no portion of the root system was resampled. To ensure that destructive sampling did not affect root exudation, samples for stem water potential (and subsequent branchlet NSC analysis from the same sample) were taken only after completion of each exudation campaign. Destructive sampling of fine-roots for NSC was conducted while locating roots for the exudation protocol but always from root sections distant from the segment used for exudation sampling.

### Eco-physiological measurements

Continuous monitoring of soil water content (SWC, %, v/v) at depth of 25 cm was obtained by Hydra Dual Depth soil moisture sensors (Soil Scout, Helsinki, Finland) starting from the onset of drought treatment. Observations were recorded hourly with one to two repeats for each treatment. To measure SWC for all saplings at pre-drought, drought and post-drought, we used the portable TDR-SDI-12 soil moisture sensor (Acclima Inc., Meridian, ID, USA; *n* = 8).

Soil properties including pH, electrical conductivity (EC), nitrate (NO₃^−^) and soil organic C (SOC) were measured on soil samples collected during drought and post-drought periods (*n* = 6). For pH, EC and NO_3_^−^ measurements, 8 g of air-dried, sieved (<2 mm) soil was shaken overnight with 50 mL of deionized water and subsequently filtered. Soil pH and EC were measured using a pH meter (Orion Lab Star pH 11, Thermo Fisher, Waltham, MA, USA) and a conductivity meter (4510Conductivity Meter, JENWAY, Cole-Parmer, Vernon Hills, IL, USA), respectively. Nitrate concentrations were determined using a Gallery Plus Discrete Analyser (Thermo Fisher). Soil organic C was determined using the loss-on-ignition (LOI) method (Ben-Dor and Banin 1989) after fractionating the soil into mineral-associated organic matter and particulate organic matter following Cotrufo et al. (2019). Organic C was estimated from organic matter content using a conversion factor of 0.58 (average C content in soil organic matter), and SOC was calculated as the sum of the organic C in the two fractions. Results of soil properties are displayed in Table S2 available as Supplementary Data at *Tree Physiology* Online.

Stem diameter and height were recorded bi-weekly for all trees during the course of the experiment (*n* = 8). Percentage loss of diameter (PLD) for the drought treatment was calculated as PLD (%) = 100 × ((*D*_max_ − *D*_min_)/*D*_max_), where *D*_max_ and *D*_min_ are the maximum and minimum diameters, respectively, before rewetting (Andriantelomanana et al. 2024). Biomass of branches and needles, stem and roots were determined at the end of the experiment for all 32 saplings (*n* = 8). The whole root system was separated from the soil and washed to remove attached particles. Each component was dried at 60 °C for at least 3 days to measure the biomass.

Rates of net leaf assimilation (A, μmol CO_2_ m^−2^ s^−1^) and stomatal conductance (gs, mol H_2_O m^−2^ s^−1^) were measured using a portable gas exchange system GFS-3000 (Walz, Effeltrich, Germany). The system was equipped with a lamp, set to a light intensity of 700 μmol m^−2^ s^−1^. Air temperature and humidity were set to ambient conditions. The flow rate was set to 750 μmol s^−1^, and impeller speed was set to step 7 (2.2 m s^−1^) inside the sample cuvette. CO_2_ was maintained at 400 p.p.m. across all treatments to ensure uniform and random measurement conditions for all saplings on the same day, eliminating the need for continuous adjustment of CO_2_ settings between treatments. For the eCO_2_ treatment, measurements were adjusted using an empirically derived correction factor. Specifically, 13 pines grown under eCO_2_ were measured sequentially with the cuvette set to 400 p.p.m. and then to 850 p.p.m. CO_2_, during the experiment. The correction factor was calculated as the ratio between assimilation at 850 and 400 p.p.m. for each plant, resulting in correction factors of 2.36 ± 0.66 for assimilation and 1.08 ± 0.30 for stomatal conductance_._ Additionally, gas exchange rates were calibrated to the actual leaf area by determining the projected leaf area using Easy Leaf Area (Easlon and Bloom 2014). Gas exchange measurements were also used to calculate intrinsic water-use efficiency (iWUE) at the leaf level, defined as iWUE = A/gs (μmol CO_2_ mol^−1^ H_2_O). Measurements were conducted on the youngest fully mature needles at midday (11:00–13:00 h) at pre-drought (DRD −38), drought (DRD 23) and recovery (DRD 57/58; *n* = 6–8), and were used for the main statistical analysis. In addition, gas-exchange measurements were taken on DRD −171, −136, −111, −81, −3/−2, 11/13, 23, 40/41, 47/48, 57/58 and were used for separate analysis (*n* = 1–8; Figure S3 available as Supplementary Data at *Tree Physiology* Online). To calculate percent exudation per assimilation at whole sapling scale, assimilation measured on leaf level and root exudation measured on fine roots were up-scaled to the whole-sapling scale (methodological details, see in supplementary methods).

Stem water potential (*Ψ*_x_) was measured after each exudation campaign on terminal branchlets containing needles that were covered for at least 20 min with tin-foil-sealed bags to allow for equilibration between needles and stem water potential. The branchlets were collected from the trees at midday (11:00–13:00 h) and directly measured. *Ψ*_x_ measurements were conducted using a Scholander-type (Scholander et al. 1965) pressure chamber (PMS Instrument Company, Albany, OR, USA) at pre-drought (DRD −37), drought (DRD 22) and post-drought (DRD 62/64; *n* = 8).

### Non-structural carbohydrate analysis

Soluble sugars and starch content were determined for branchlets and fine roots (<2 mm) at pre-drought, drought and post-drought (*n* = 7–8). Branchlet NSC comprised sapwood and bark. Exact sampling dates for branchlet NSC correspond to the *Ψ*_x_ measurements and for fine roots to the root exudation campaigns as mentioned above. Extractions were conducted following Landhäusser et al. (2018) using 30 ± 1 mg of dried sample, except for some fine-root samples, for which smaller amounts (≥15 mg) were used due to limited material. Soluble sugars were extracted in ethanol and quantified as glucose and fructose by ultra-high-speed liquid chromatography (UFLC) system (Prominence UFLC, Shimadzu Scientific Instruments, Kyoto, Japan). Starch was measured colorimetrically as glucose after enzymatic hydrolysis. Sampling and NSC assay reproducibility were monitored using internal standards (internal reference sample, starch standard and HPLC standard), which were routinely measured between samples to ensure the quality and consistency of the analysis. Results are expressed as % (w/w) relative to dry matter. For methodological details, see Supplementary Methods.

### Root exudation collection

Root exudates were collected from intact fine roots using a modified non-soil syringe system, based on Phillips et al. (2008). Exudation campaigns started on days DRD −61 and − 47 (pre-drought), DRD 17 (drought) and DRD 59 (post-drought; *n* = 8 trees). The pre-drought campaign was conducted on two different days due to logistical constraints; trees were randomly selected across treatments and rooms, and exudation rates did not differ between the two sampling campaigns. Fine roots remained attached to the trees throughout the procedure. For each tree, root exudates were collected from two fine-root segments (technical replicates), providing within-tree subsamples for exudation measurements. Roots were gently cleaned using a sterile C-free nutrient solution (0.5 mM NH₄NO₃, 0.1 mM KH₂PO₄, 0.2 mM K₂SO₄, 0.2 mM MgSO₄, 0.3 mM CaCl₂) and placed back into the pot soil to allow recovery from disturbance for ~15 h. Next, roots were rinsed again and were placed into acid-washed 30 mL glass syringes filled with 0.5–1.3 mm acid-washed glass beads and 10 mL of the C-free nutrient solution (moisture of the syringes was ~21%). Syringes were wrapped in aluminum foil to block light. After a 48-h incubation period, the nutrient solution was recovered by flushing each syringe with an additional 10 mL of double-distilled water. Four blank syringes (syringes without root) per campaign were processed identically, and blank C values were subtracted from the samples. On average, C concentrations in root-containing syringes were 1.6-fold higher than in blanks. Exudate solutions were immediately filtered through 0.22 μm sterile syringe filters (Millex PVDF, Millipore Co., Billerica, MA, USA), split into aliquots for Total organic carbon (TOC) and metabolomic analysis and stored at −20 or −80 °C, respectively. Total organic carbon (TOC) content was quantified using a TOC analyzer (Aurora 1030 W TOC Analyzer with a 1088 rotary TOC auto sampler; OI Analytical, TX, USA). The root exudation rate was calculated as the total recovered C per root surface area, averaging the two technical replicates per tree (μg C cm^−2^ day^−1^). The root surface area was measured after exudate collection using a flatbed scanner (HP Color Jet Pro MFP M477fdn) and analyzed with RhizoVision Explorer 2.0.3 software. Roots were then oven-dried (48 h at 60 °C) and weighed to determine root biomass.

We used the first rather than the second flush (48 vs 96 h after transfer of roots to syringes, respectively) in our phosphorus-poor Mediterranean forest soil, following the recommendation of our previous study (Obersteiner et al. 2026*a*) to minimize root acclimation to the phosphorus-containing incubation solution. Consistent with Obersteiner et al. (2026*a*), exudation rates of non-stressed roots were higher in the second flush (Table S3 available as Supplementary Data at *Tree Physiology* Online). By contrast, exudation rates of drought-stressed roots decreased in the second flush, likely due to osmotic adjustment; however, rates remained significantly higher than those of non-stressed roots (Table S3 available as Supplementary Data at *Tree Physiology* Online). Because the effect of the incubation solution on stressed roots remains uncertain in our system, we proceeded with the first flush.

### Metabolite analysis of root exudates using LC–MS

A subset of the collected samples was used for metabolomic analysis (*n* = 5): root exudate samples at pre-drought, drought and post-drought and two blank samples (samples without a root) were lyophilized and analyzed for polar and semi-polar metabolites using Liquid Chromatography coupled to high-resolution mass spectrometry (LC-HRMS) at MS-Omics (Vedbæk, Denmark), as described in Obersteiner et al. (2026*b*). Peaks were aligned and integrated using Compound Discoverer 3.3 and Skyline 23.1. Compounds were annotated at four confidence levels based on retention time, accurate mass and MS/MS spectra; only Level 1 (retention time matched to in-house standards, accurate mass and MS/MS spectra) and Level 2a (retention time matched to in-house standards and accurate mass) annotations were retained for further analysis. Peak areas were normalized by root dry weight (Williams et al. 2022) to compare treatment effects on the amount of each metabolite exuded per unit biomass. Metabolites with levels lower than the blanks in >33% of sample groups were excluded from further analysis. Carbon-to-nitrogen (C:N) ratios were calculated from the molecular formula of each identified metabolite. For methodological details, see Supplementary Methods.

### Metabolic pathway analysis

Metabolites identified in root exudates that increased significantly in aCO_2_-DS and/or eCO_2_-DS during the drought phase (total of 54 metabolites) were uploaded to MetaboAnalyst 5.0 (www.metaboanalyst.ca) for pathway analysis, using *Arabidopsis thaliana* libraries. The over-representation analysis method was hypergeometric test, and the node importance measure was relative betweenness centrality (see Table S4 available as Supplementary Data at *Tree Physiology* Online).

### Statistical analysis

Data were analyzed and visualized using R version 4.4.0 (Team 2024b) and its interface RStudio (Team 2024a). Unless otherwise stated, all plots were generated using ‘ggplot2’ (Wickham 2016). Biomass components (needle, branch, stem and root), measured at the end of the experiment, were analyzed with linear models (LMs; ‘stats’ package), with drought, CO_2_ and their interaction as fixed effects. Fixed effects were assessed using Type III *F*-tests. Traits measured repeatedly over time at pre-drought, drought and post-drought (stem diameter, assimilation, soluble sugar and starch) were analyzed with linear mixed-effects models (LMMs; ‘lmerTest’; Kuznetsova et al. 2017), with drought, CO_2_, time and their interactions as fixed effects, and tree identity included as a random factor to account for repeated measurements of the same individual tree. Fixed effects were assessed using Type III F-tests with Satterthwaite-approximated denominator degrees of freedom. For stem diameter, the last measurement of each experimental phase was used for analysis. Because root exudation data were right-skewed and included exact zeros, root exudation was analyzed separately using a generalized linear mixed model fitted with a Tweedie distribution and log link (‘glmmTMB’; Brooks et al. 2017). This model included drought, CO_2_, time and all their interactions as fixed effects, with tree identity included as a random factor. Fixed effects were assessed using Wald chi-square tests. When a significant interaction involving experimental phase was detected, follow-up tests based on the same full model were used to evaluate the relevant CO_2_ and drought effects and their interaction within each phase. We also assessed whether the drought response differed among phases separately within each CO_2_ level and compared drought-stressed with well-watered trees within each phase and CO_2_ level. These analyses were performed using estimated marginal means (‘emmeans’; Lenth 2026). *P*-values were Holm-adjusted within each response and contrast family. Overall model results are presented in Table S5, and follow-up tests in Table S6 (available as Supplementary Data at *Tree Physiology* Online).

All models were fitted to available observations without imputation. Model assumptions were verified by inspecting residual plots for homoscedasticity and normality. When necessary, variables were transformed prior to analysis (net photosynthesis: log10 [x − min(x) + 1]; starch log10 [x]). Data are presented as mean ± standard deviation (SD), except in figures, where we show the standard error (SE).

Changes in metabolite compositions were visualized as log_2_ fold-changes (FCs), calculated as the mean of biological replications (*n* = 5) of the treatment groups (aCO_2_-DS, eCO_2_-WW, eCO_2_-DS) divided by the control group (aCO_2_-WW) for each sampling point. Only metabolites that increased by more than double (log_2_ FC > 1) or decreased by more than half (log_2_ FC < −1) and showed statistically significant changes were presented. The purpose of FC analysis was to compare absolute value change between two group averages and find some metabolites that changed (i.e., up-regulated or down-regulated) in treatment groups compared with the control. For significance testing, the Box–Cox transformation was applied to unlogged data to stabilize variance prior to testing. Significant differences between the groups were then determined by *t*-tests (‘stats’ package) with Bonferroni correction (adjusted *P*-value <0.05). For log_2_-transformed peak areas of individual samples without FC, see Figure S4 available as Supplementary Data at *Tree Physiology* Online. The log_2_-transformed FCs were visualized as a heatmap using ‘ComplexHeatmap’ (Gu et al. 2016). Principal component analysis (PCA) was generated with the complete metabolite dataset. Significant differences between groups in the PCAs were determined by Permutational Multivariate Analysis of Variance (PERMANOVA) on a Bray–Curtis distance matrix using ‘vegan’ (Oksanen et al. 2026).

Effect sizes were calculated between the control group (aCO_2_-WW) and each of the three treatment groups (aCO_2_-DS, eCO_2_-WW and eCO_2_-DS) as Cohen’s *d* (‘effsize’; Torchiano 2020) for the following measured variables: biomass (aboveground = stem + needles + branches; belowground = roots), diameter, photosynthesis, soluble sugars and starch (above- and belowground) and root exudation. To allow for effect size estimation and multivariate analysis (PCA), missing values were replaced with the mean of the corresponding treatment group, because such analyses require a complete data matrix. This simple imputation method was chosen because the number of missing values was low (<2%). The imputation did not influence the direction of the results but did influence statistical significance in some comparisons. Results from a complete-case analysis (excluding all rows with missing data) are provided in Figure S5 available as Supplementary Data at *Tree Physiology* Online. Significant differences between groups were determined based on the confidence interval for Cohen’s *d*. The same variables used for calculating the effect size were used in the PCA. Significant differences between groups in the PCAs were determined by PERMANOVA as described above.

### Limitations of the study

First, measuring root exudation under drought conditions presents unique challenges with any currently available method, as it usually implies altering the moisture level (Brunn et al. 2025). In this study, we employed the commonly used soil-syringe method (Phillips et al. 2008), in line with other drought studies in trees (Jakoby et al. 2020, Brunn et al. 2025, W.Li et al. 2025), in which roots are incubated at higher moisture levels. The extent to which the moisture level alters exudation behavior of drought-stressed saplings is not fully known. A substantial fraction of primary metabolites can leave roots by passive pathways (simple diffusion or diffusion via uniport), whereas secondary metabolites in root exudates are predominantly released via active transporters (Canarini et al. 2019). Hence when membrane permeability is increased by drought due to increased oxidative damage, passive leakage is possible especially for smaller compounds, e.g., sugars, certain amino acids and organic acids. Hence, although we cannot rule out increased passive leakage of primary exudates from drought-stressed roots, the observed increase in phenolics, purines and terpenoids, especially under drought indicates physiological responses rather than artefacts. Moreover, the positive correlation between soluble sugar concentrations in fine roots and exudation rates further reinforces that higher exudation reflected increased available C in roots rather than an artifact of wet collection conditions.

Second, although we used acid-washed glass syringes and rinsed roots with sterile C-free nutrient solution, a negligible contribution from root-associated bacteria remaining on the root during insertion (via consumption of exudates or release of bacterial metabolites) is possible. However, because the metabolite profiles were dominated by compounds and patterns typical of plant stress responses, we are confident that metabolites shown in this study were plant derived. We also accounted for background C contamination by subtracting blanks from samples. In the metabolite dataset, we excluded 56 compounds that were more abundant in blanks than in samples, suggesting that some background C compounds may have been taken up by roots during incubation. Thus, while these metabolites were removed from further analysis, we cannot fully exclude metabolite reuptake more broadly, and some signals may have been underestimated or missed.

Lastly, although saplings were grown in relatively large 25 L pots, root systems remained confined, showing an average density of 5.6 ± 1.4 g root L^−1^, exceeding the recommended 1 g root L^−1^ (Poorter et al. 2012), potentially limiting growth. While this confinement exceeds the general threshold recommended for pot experiments to prevent growth limitation, it remains well below the root biomass densities of ~11 g root L^−1^ commonly observed in natural montane shallow-soil pine stands (~15 cm depth) with ~500 trees ha^−1^; Rog et al. 2021). Thus, while pot volume constraints must be acknowledged, the root density within our experimental setup stayed within realistic boundaries compared with field-grown counterparts and did not distort our comparative treatment dynamics. Overall, we are confident that these limitations did not affect the main patterns observed in this study, as we addressed them conservatively. While they may have influenced absolute exudation levels (shifting the baseline slightly higher or lower), the treatment-driven differences in exudate profiles and rates appear robust.

## Results

### Tree growth

**Figure 2:**
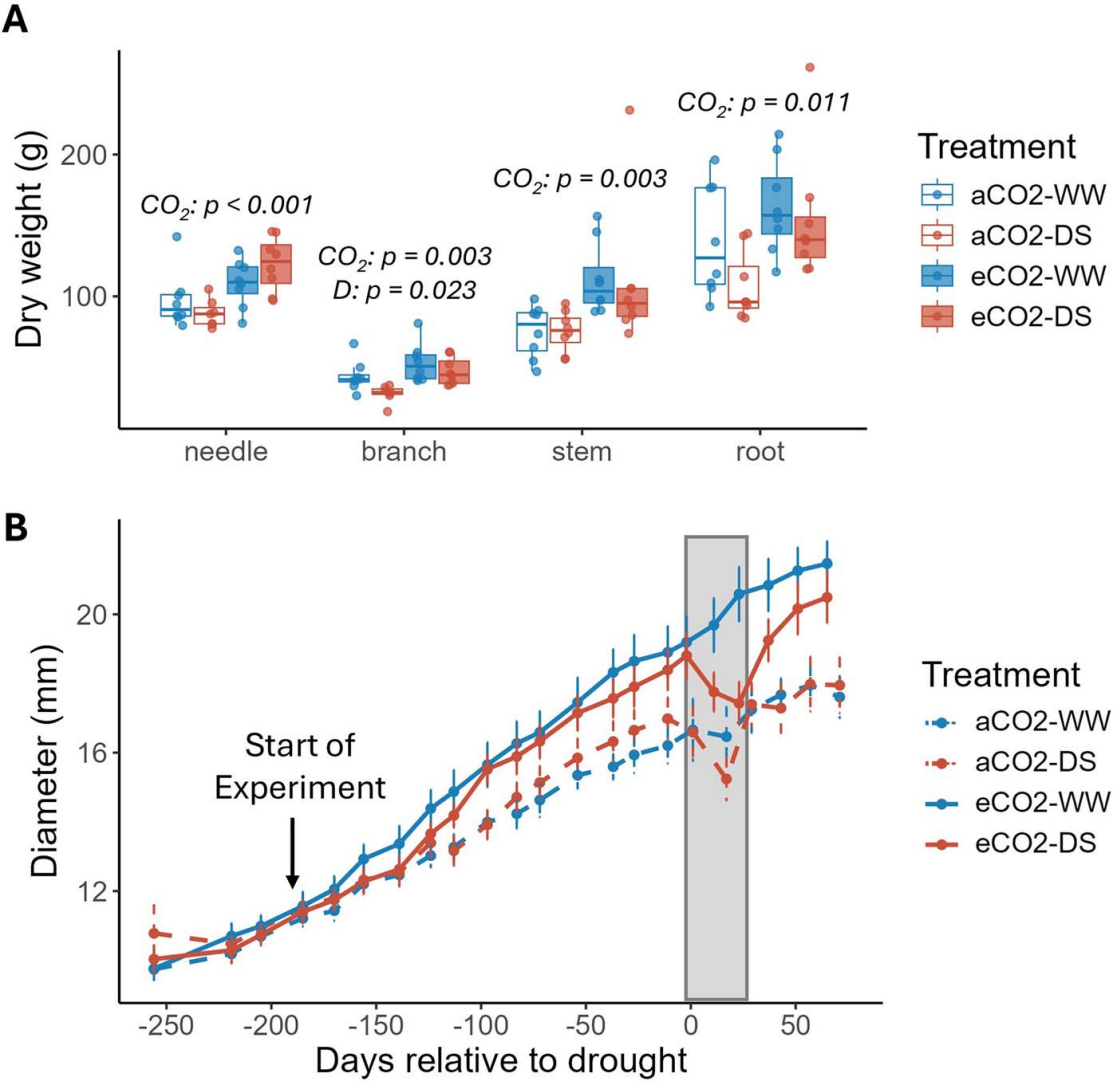
Dry weight (g) of needle, branch, stem and root tissues of pines under single and combined CO_2_ and drought treatment at the end of the experiment (DS = drought-stressed, WW = well-watered; A). Lines in boxes represent the median, the top and bottom of boxes represent first and third quartiles and whiskers represent the 1.5 interquartile range. Stem diameter of pines measured over the period of the experiment under single and combined CO_2_ and drought treatment (B). Means of stem diameter are depicted with standard error bars. Gray background indicates the drought period. *P*-values for significant treatment effects are shown in (A) (D = drought treatment; *n* = 8).

Tree growth in eCO_2_ treatment was significantly higher in comparison to the aCO_2_ for all tree organs, needles, branches, stems and roots (Figure 2A). Drought treatment (aCO_2_-DS + eCO_2_-DS) reduced biomass of branches compared with well-watered trees (aCO_2_-WW + eCO_2_-WW; *F*_1,27_ = 5.84, *P* = 0.023). Well-watered pines under eCO_2_ reached the highest total biomass with 469.7 ± 24.8 g while the lowest biomass was of aCO_2_-DS with 331.4 ± 18.7 g. Initial stem diameter was on average 10.07 ± 1.35 mm. Stem diameter was significantly affected by CO_2_, time, CO_2_ × time and drought × time (Table S5 available as Supplementary Data at *Tree Physiology* Online). Overall, stem diameter was greater under eCO_2_ compared with aCO_2_ (Figure 2B, *F*_1, 28_ = 17.81, *P* < 0.001). Pines grown under eCO_2_ reached on average 21.0 ± 0.5 mm, compared with 17.8 ± 0.5 mm under aCO_2_, at the end of the experiment (Figure 2B). During drought exposure (DRD 0–24), stems were shrinking and the PLD was 10.3% and 7.3%, in the aCO_2_-DS and eCO_2_-DS group, respectively. After re-irrigation, stem diameter increased, reaching a total increase of 15.7% and 12.2% relative to the minimum diameter in the aCO_2_-DS and eCO_2_-DS group, respectively_._ Over the course of the experiment, tree height increased on average from 104.21 ± 14.33 cm to 111.32 ± 14.45 cm, with no significant differences among treatments (Figure S6 available as Supplementary Data at *Tree Physiology* Online). Hence, we attribute the higher stem biomass under eCO_2_ to the higher radial growth under eCO_2_. For the full overall and interaction effects, see Table S5 available as Supplementary Data at *Tree Physiology* Online.

### Root exudation and CO_2_ assimilation

**Figure 3:**
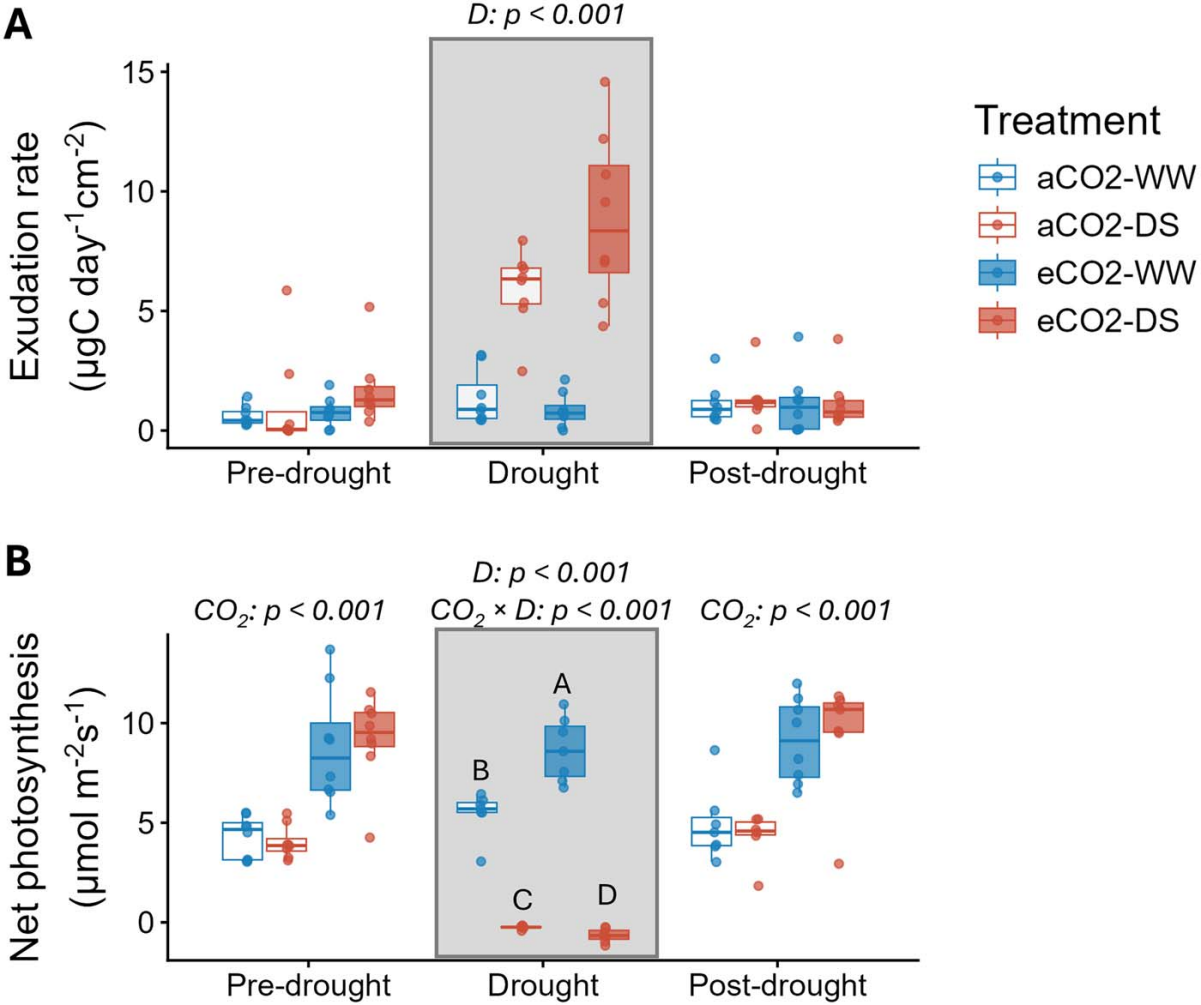
Root exudation rate (μg C day^−1^ cm^−2^; *n* = 8; A), net photosynthesis (μmol m^−2^ s^−1^, *n* = 6–8; B) of pines at pre-drought, drought (gray background) and post-drought under single and combined CO_2_ and drought treatment (DS = drought-stressed, WW = well-watered). Lines in boxes represent the median, the top and bottom of boxes represent first and third quartiles and whiskers represent the 1.5 interquartile range. Significant *P*-values from phase-specific simple-effect tests are shown (D = drought treatment). Letters indicate holm-adjusted pairwise treatment differences where CO_2_ × drought was significant (*P* < 0.05).

Root exudation was significantly affected by drought, time and their interaction, whereas neither CO_2_ nor any interaction involving CO_2_ was significant (Table S5 available as Supplementary Data at *Tree Physiology* Online). Root exudation did not differ significantly among treatments during the pre- or post-drought phases; however, during the drought phase, it increased strongly in drought-stressed trees (Figure 3A). Relative to their respective well-watered controls, drought-stressed pines exuded 4.3-fold more under aCO_2_ and 10.4-fold more under eCO_2_, reaching 5.90 ± 1.64 and 8.86 ± 3.52 μg C day^−1^ cm^−2^, respectively. Exudation was 50% higher in eCO_2_-DS than in aCO_2_-DS, but this difference was not statistically significant. The drought response varied significantly over time under eCO_2_ (χ^2^_2_ = 17.56, *P* < 0.001), whereas the corresponding interaction under aCO_2_ was marginal but nonsignificant (χ^2^_2_ = 5.96, *P* = 0.051; Table S6 available as Supplementary Data at *Tree Physiology* Online). This pattern suggests a more pronounced drought response under eCO_2_ but does not demonstrate that eCO_2_ strengthened the effect of drought.

Photosynthesis was significantly affected by CO_2_, drought and time and their interactions but not by the CO_2_ × drought interaction (Table S5 available as Supplementary Data at *Tree Physiology* Online). At pre-drought, assimilation rates were 4.16 ± 0.90 and 8.98 ± 2.44 μmol m^−2^ s^−1^, under aCO_2_ and eCO_2_, respectively (Figure 3B), showing a 115% increase under eCO_2_ matching the expected eCO_2_/aCO_2_ ratio of 850/400 p.p.m. By the 24^th^ day of drought, the photosynthesis rate of drought-exposed pines declined below zero in both treatments and was lower in eCO_2_-DS (−0.65 ± 0.34 μmol m^−2^ s^−1^) than in aCO_2_-DS (−0.25 ± 0.08 μmol m^−2^ s^−1^), indicating higher CO_2_ respiration than assimilation. Photosynthesis in drought-stressed trees had already declined to near zero by DRD 12/14 (0.35 ± 0.24 μmol m^−2^ s^−1^; Figure S3A available as Supplementary Data at *Tree Physiology* Online). At post-drought, no difference was found between previously drought stressed trees and well-watered trees (aCO_2_-WW vs aCO_2_-DS and eCO_2_-WW vs eCO_2_-DS), indicating complete recovery of photosynthesis.

Across all measured time points, iWUE was 117.6% higher under eCO_2_ than under aCO_2_ (116.24 ± 60.79 vs 53.33 ± 35.18; *t*_29.3_ = −7.32, *P* < 0.001; Figure S3C available as Supplementary Data at *Tree Physiology* Online). During pre-drought and post-drought conditions, root exudation relative to assimilation at the tree scale averaged 2.2 ± 3.6% and 2.7 ± 2.9%, respectively, with no significant differences between treatments (Figure S7A available as Supplementary Data at *Tree Physiology* Online). However, during drought exposure, when assimilation dropped below zero (DRD 24), the exudation-to-assimilation ratio approached ‘infinity’, indicating that drought-stressed saplings continued to exude C despite the absence of new C assimilation. This pattern was also reflected in the exudation per biomass, where during drought exposure, drought-stressed trees reached values of 0.037 ± 0.018%, while well-watered trees only reached 0.007 ± 0.006% (Figure S7B available as Supplementary Data at *Tree Physiology* Online).

### Metabolite composition of root exudates

**Figure 4:**
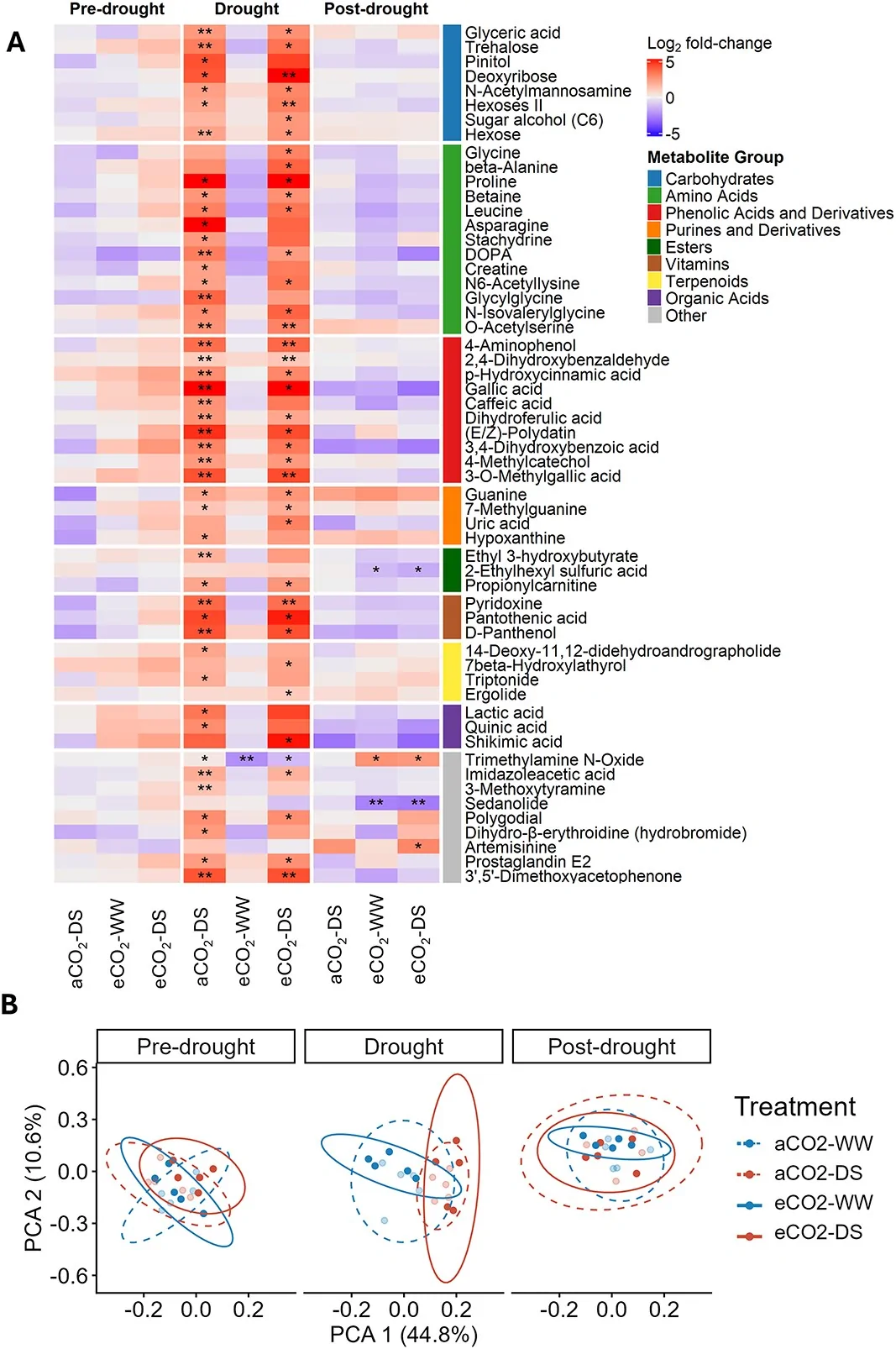
Metabolic profiles of root exudates of pines at pre-drought, drought and post-drought presented in a heatmap (A) and PCA (B). Values in the heatmap are shown as log_2_-transformed fold-change (FC) of each treatment group compared with the control group (DS = drought-stressed, WW = well-watered). Only metabolites with a FC >1 or <−1 that changed significantly are presented. A *t-*test with Bonferroni correction for multiple comparisons was used to assess the differences between control and treatment groups. The *P*-value threshold for significance was set at *P* < 0.05 (^*^) and 0.01 (^**^) (*n* = 5).

LC-MS metabolite profiling resulted in the annotation of a total of 196 metabolites: 42 polar metabolites and 154 semi-polar metabolites (certainty levels 1 and 2a; see Materials and methods). Among these 196 metabolites and after excluding metabolites with levels lower than the blanks, 57 metabolites exhibited significant changes in at least one treatment, compared with the control (aCO_2_-WW; Figure 4A). These compounds were classified into nine chemical groups: carbohydrates (comprising 14.0% of significant metabolites), amino acids (22.8%), phenolic acids and derivatives (17.5%), purines and derivatives (7.0%), esters (5.3%), vitamins (5.3%), terpenoids (7.0%), organic acids (5.3%) and others (15.8%).

Overall, the PCA showed clear clustering of drought treatment, however no clear clustering of eCO_2_ treatment at any time point (Figure 4B). The explained variance for PCA1 is 44.8%, while PCA2 explains 10.6% of the variance. Drought treatment had a significant effect on metabolite composition and even stronger as interactive effect with time (PERMANOVA drought: *F*_1,48_ = 5.51, *P* = 0.008; drought × time: *F*_2,48_ = 5.98, *P* = 0.001), whereas interestingly CO_2_ treatment did not show any significant effect on metabolite composition as single effect or interactive effect with drought and/or time (Table S7 available as Supplementary Data at *Tree Physiology* Online).

No metabolites showed significant fold changes relative to aCO_2_-WW under pre-drought conditions (Figure 4A). During drought exposure (DRD 0–24), 54 metabolites changed significantly, of which 26 were nitrogenous and 28 were non-nitrogenous. In aCO_2_-DS, 47 metabolites increased significantly in the aCO_2_-DS group. These included 11 amino acids, 7 carbohydrates, 10 phenolic acids and derivatives, 3 purines and derivatives, 3 vitamins, 2 organic acids, 2 terpenoids, 2 esters and 7 classified as ‘others’. Of these, 32 metabolites also increased significantly under eCO_2_-DS, while 7 metabolites increased only in eCO_2_-DS (Sugar alcohol, Glycine, beta-Alanine, Uric acid, 7-beta-Hydroxylathyro, Ergolide and Shikimic acid; Figure S8 available as Supplementary Data at *Tree Physiology* Online). The 10 metabolites with the highest log_2_ FC were: Lactic acid, Proline, Asparagine, Pantothenic acid, Gallic acid, Pinitol, (E/Z)-Polydatin, Deoxyribose, Shikimic acid and 3-O-Methylgallic acid; all increased in the aCO_2_-DS and/or eCO_2_-DS. Overall, drought caused a broadly similar metabolite increase under both CO_2_ levels, although Trimethylamine N-Oxide decreased under eCO_2_-WW and eCO_2_-DS.

After re-irrigation, most drought-responsive metabolites returned to irrigated levels. However, Artemisinine increased under eCO_2_-DS, showing the same trend under aCO_2_-DS at post-drought. Further, Trimethylamine N-Oxide increased, while 2-Ethylhexyl sulfuric acid and Sedanolide decreased in both eCO_2_-WW and eCO_2_-DS at post-drought.

Pathway enrichment analysis of metabolites that increased in drought-stressed saplings revealed significant enrichment of glycine/serine/threonine metabolism, the one-carbon pool by folate and β-alanine metabolism (Table S4 available as Supplementary Data at *Tree Physiology* Online), despite the small number of metabolites assigned to each pathway. These enrichments were driven by glycine and D-glycerate, glycine and betaine and β-alanine and pantothenate, respectively.

### Soluble sugars and starch content

**Figure 5:**
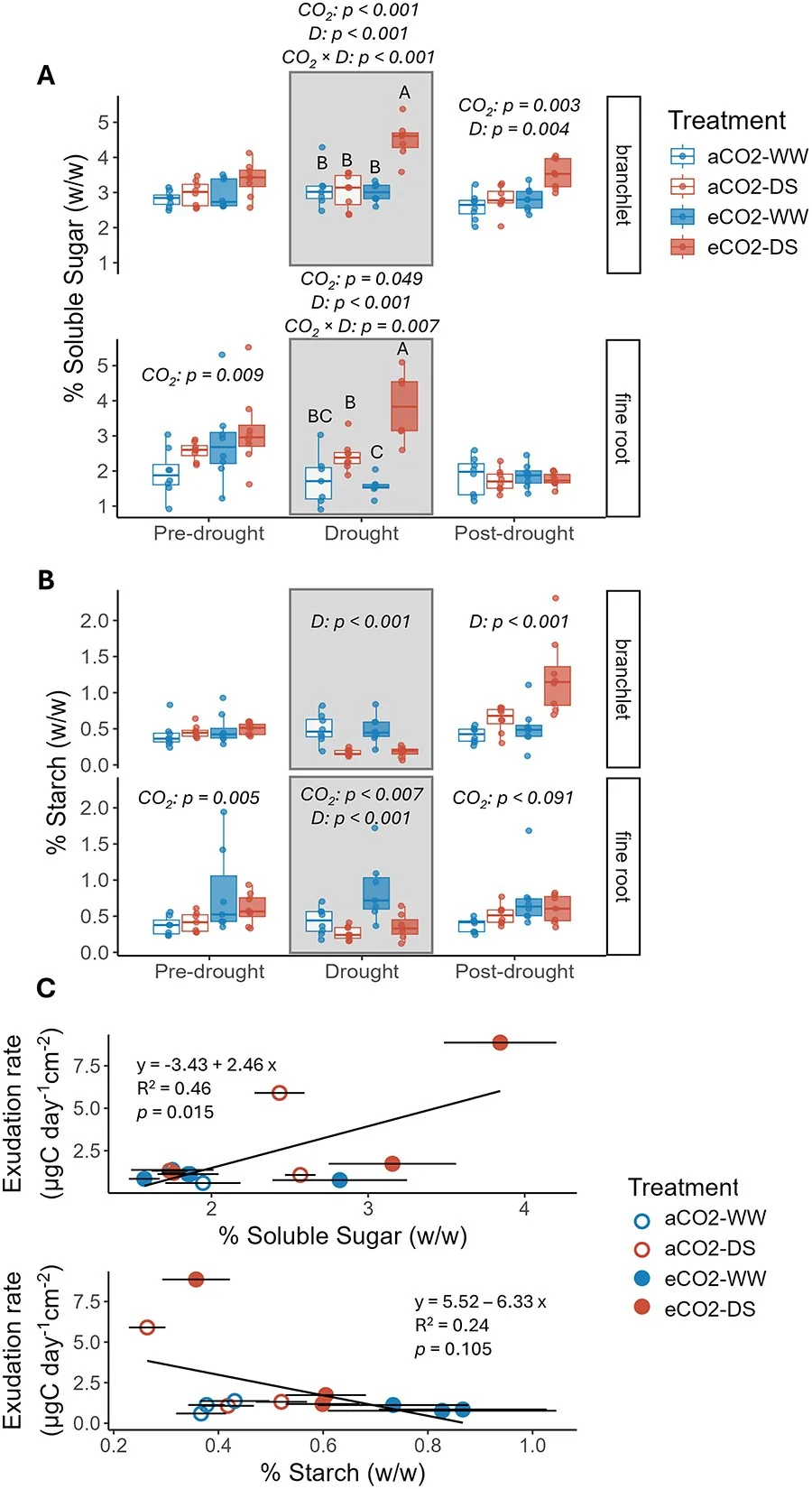
Percent of soluble sugar (glucose and fructose; w/w; A) and starch (w/w; B) of branchlets and fine roots of pines at pre-drought, drought (gray background) and post-drought under single and combined CO_2_ and drought treatment (DS = drought-stressed, WW = well-watered; *n* = 6–8). Lines in boxes represent the median, the top and bottom of boxes represent first and third quartiles and whiskers represent the 1.5 interquartile range. Significant *P*-values from phase-specific simple-effect tests are shown (D = drought treatment). Letters indicate Holm-adjusted pairwise treatment differences where CO_2_ × drought was significant (*P* < 0.05). Correlation of root exudation rate with percent soluble sugar and starch in fine roots (w/w; C).

Soluble sugar content showed the strongest treatment response during the drought phase, where values in eCO_2_-DS were 38% and 52% higher than in aCO_2_-DS in branchlets and fine roots, respectively, reaching the highest values of 4.2 ± 1.0% w/w and 3.7 ± 1.0% w/w (Figure 5A). CO_2_, drought, time and their three-way interaction significantly affected soluble sugars in the branchlets and roots. In addition, branchlets showed a significant CO_2_ × drought interaction, while roots showed a significant drought × time interaction (Table S5 available as Supplementary Data at *Tree Physiology* Online). Follow-up tests showed that the drought response varied significantly over time under eCO_2_, but not under aCO_2_, in both tissues (Table S6 available as Supplementary Data at *Tree Physiology* Online), indicating that eCO_2_ amplified drought-associated soluble sugar accumulation. Importantly, the interaction contrasts accounted for the higher values at pre-drought in eCO_2_-DS and showed that the separation between drought and control trees increased significantly from pre-drought to drought under eCO_2_ in both branchlets (*t*_54.6_ = 3.76, *P* = 0.001) and fine roots (*t*_55.7_ = 4.22, *P* < 0.001).

Interestingly, starch content in roots was more strongly affected by the treatments than in branchlets, with significant effects of CO_2_, drought, CO_2_ × drought, time and drought × time, whereas in branchlets only time and drought × time were significant (Table S5 available as Supplementary Data at *Tree Physiology* Online). Starch content declined under drought in both branchlets and fine roots, decreasing from 0.49 ± 0.19 to 0.18 ± 0.06% w/w in branchlets and from 0.63 ± 0.39 to 0.31 ± 0.14% w/w in fine roots (Figure 5B; Table S6 available as Supplementary Data at *Tree Physiology* Online). Across the experimental period, fine-root starch content was higher under eCO_2_ than under aCO_2_, although the magnitude of this effect depended on drought treatment, as indicated by the significant CO_2_ × drought interaction (Table S6 available as Supplementary Data at *Tree Physiology* Online). Exudation rate and soluble sugar content in the fine roots, which were consistently sampled 3 days apart, showed a positive correlation of *R*^2^ = 0.46 (*P* = 0.015); however, no correlation was found with the starch content (*R*^2^ = 0.24, *P* = 0.105; Figure 5C). Note, however, that the correlation with soluble sugar was mainly driven by eCO_2_-DS during the drought phase (see sensitivity analysis in Figure S9 available as Supplementary Data at *Tree Physiology* Online).

### Effect size and PCA

**Figure 6:**
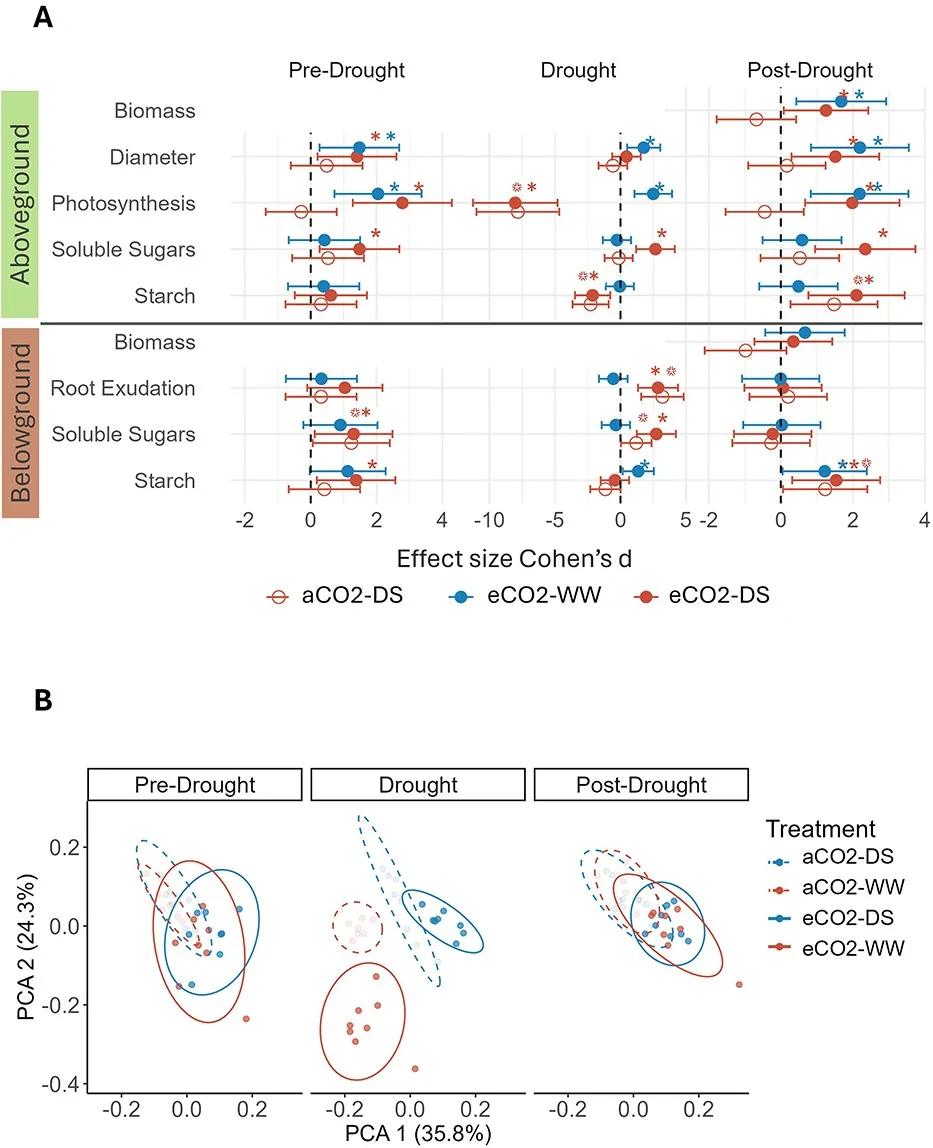
Cohen’s *d* effect sizes for pairwise comparisons of each treatment group compared with the control group at pre-drought, drought and post-drought (DS = drought-stressed, WW = well-watered; A) Error bars represent 95% confidence intervals of the effect sizes; asterisks indicate significant differences from the control. PCA at pre-drought, drought and post-drought under single and combined CO_2_ and drought treatment including all variables shown in Panel A (B).

Overall, we found a positive effect of eCO_2_ on the aboveground biomass, diameter and photosynthesis (Figure 6A). The eCO_2_-WW and aCO_2_-DS group showed higher soluble sugar content in the branchlets throughout the experiment, with fine roots reflecting similar trends under pre-drought and drought. Starch content in drought-stressed trees decreased in the branchlets, while soluble sugars in fine roots remained elevated at drought time point. Root exudation increased in drought-stressed trees regardless of CO_2_ levels at drought time point. The PCA of all measured variables, excluding metabolites, showed separation by CO_2_ treatment across the experiment and clear separation by drought treatment at the drought phase, with the strongest visual separation occurring between aCO₂-DS and eCO₂-DS (Figure 6B). PCA1 and PCA2 explained 35.8% and 24.3% of the variation, respectively. Overall, CO_2_ and drought treatment had significant effects on measured variables (PERMANOVA CO_2_: *F*_1,84_ = 60.42, *P* = 0.001; drought: *F*_1,84_ = 7.32, *P* = 0.006; Table S8 available as Supplementary Data at *Tree Physiology* Online), whereas their interaction was not significant.

## Discussion

eCO_2_ resulted in increased net photosynthesis and biomass of needles, branches, stems and roots in young pine trees (Figures 2 and 3, and Figure S3A available as Supplementary Data at *Tree Physiology* Online). Overall, the rate and composition of root exudates showed no response to eCO_2_ (Figure 3). When the trees experienced drought stress, which reduced their hydraulic and photosynthetic activities, root exudation strongly increased (Figure 3 and Figure S2 available as Supplementary Data at *Tree Physiology* Online). Moreover, during the drought phase, soluble sugars in the branchlets and fine roots increased in eCO_2_-DS, while starch levels of drought-stressed trees decreased in branchlets and fine roots (Figure 5). The metabolite composition of root exudates showed a strong drought response under aCO_2_ and eCO_2_: drought conditions led to an increase mainly in phenolic acids (e.g., gallic acid and caffeic acid), amino acids and key osmoprotectants such as trehalose, proline and betaine (Figure 4A).

### Effect of single treatment of eCO_2_ and drought on C allocation

Our results confirm our hypothesis about the effect of eCO_2_ on C allocation to growth, showing that increased assimilation under eCO_2_ led to increased above- and belowground biomass as previously documented in young forest stands (Leuzinger and Hättenschwiler 2013, Walker et al. 2019). In contrast, root exudation did not increase under eCO_2_, contrary to our expectations from former studies in forest trees (Phillips et al. 2011, Ziegler et al. 2023). One likely explanation is that eCO_2_-induced increases in exudation are most pronounced when growth is constrained, often by nutrient availability, whereas under less constrained conditions additional photosynthates are preferentially invested in growth (Phillips et al. 2011, Reay et al. 2025). In our experiment, stem diameter increased continuously under eCO_2_ and did not level off (Figure 2B), suggesting no strong sink limitation, while the increase of root NSC could be interpreted as C surplus.

Interestingly, soil N did not co-vary with exudation rate across treatments (NO_3_^−^, Table S2 available as Supplementary Data at *Tree Physiology* Online). For example, NO_3_^−^ in eCO_2_-WW declined from the drought to the post-drought period, while exudation rates remained unchanged, indicating that short-term variation in mineral N availability did not directly or indirectly regulate root exudation under these conditions, as it apparently did not constrain growth sinks. Indeed, in a previous experiment using the same infrastructure, Dror and Klein (2021) also found no significant increase in root exudation in pine saplings under eCO_2_. Further, in contrast to the organic-acid enrichment frequently reported under eCO_2_ in herbaceous systems and often linked to nutrient acquisition (Zeng et al. 2024), exudate composition in our study remained unchanged under eCO_2_. This is consistent with findings in *Eucalyptus*, where organic-acid rate and composition also showed no response to eCO_2_ (Hasegawa et al. 2023).

The hypothesis that root exudation would increase under drought while growth was constrained was confirmed. This observation aligns with studies showing that trees undergoing drought stress tend to allocate a greater proportion of available C to root exudation (Brunn et al. 2022, Oppenheimer-Shaanan et al. 2022, Obersteiner et al. 2026b). In contrast, a recent study on Qinghai spruce reported a decline in root exudation with increasing drought severity (W.Li et al. 2025). This discrepancy suggests species-specific differences in drought sensitivity and C allocation strategies, with our more drought-tolerant species potentially responding differently to drought-induced reductions in assimilation and growth.

### Effect of combined drought × eCO_2_

Soluble sugars increased synergistically under drought × eCO_2_, and the drought-induced increase in root exudation tended to be more pronounced under eCO_2_. Thus, our third hypothesis—that eCO_2_ would mitigate drought-driven growth limitations and reduce root exudation—was not supported. Instead, the response tended in the opposite direction, although evidence that eCO_2_ amplified root exudation was not statistically proven. This pattern could initially be interpreted as increased C surplus resulting from drought-induced growth restriction (Figure 6A, Prescott et al. 2020). However, net photosynthesis declined sharply and eventually became negative under drought, while starch tended to decrease rather than accumulate. Therefore, this response may instead reflect osmotic adjustment and remobilization of stored C rather than a true surplus-C state (Guo et al. 2021). A similar positive correlation between root NSC and root exudation has also been reported in *Larix gmelinii* under drought (Yin et al. 2023), highlighting a similar coupling in other tree systems. Accordingly, the increased soluble sugar content may have derived partly from recent photosynthates at drought onset, and partly from the active mobilization of starch reserves to sustain root C export under drought (MacNeill et al. 2017). Conversely, Dror and Klein (2021) found only a transient effect of eCO_2_ on sugar concentration in fine roots and small branches in pines. Interestingly, Aubrey and Teskey (2018) found in pines that coarse roots alone were able to sustain starch levels in fine roots under C starvation in trenched trees. This highlights the critical role of coarse roots as a C source during periods of C starvation, a dynamic that was not directly measured in our study but may have contributed to the observed response.

Importantly, our third hypothesis was based on the often-predicted alleviation of drought stress under eCO_2_ due to increased WUE, leading to higher SWC (Morgan et al. 2004, Manderscheid et al. 2014, Paudel et al. 2018, O’Connor et al. 2024) and thereby mitigating drought-induced growth reductions (Umaña et al. 2025). However, despite slightly reduced stomatal conductance, which is not commonly observed in conifers (Klein and Ramon 2019) and increased iWUE (Figures S3B and C available as Supplementary Data at *Tree Physiology* Online), drought impacts on stem water potential and SWC were not alleviated by CO_2_ fertilization and reached similar levels as under aCO_2_ (Figure S10 available as Supplementary Data at *Tree Physiology* Online; Perry et al. 2013). The higher biomass of pines under eCO_2_ possibly led to an increase in total water demand, negating some of the benefits. Consistent with this, stem diameter dynamics showed the expected drought-induced shrinkage in both CO_2_ treatments, with PLD values indicating only a slight mitigation under eCO_2_ (Figure 2B). Hence, we conclude that drought-induced growth restriction was not alleviated in our system under eCO_2_, leading potentially to higher C surplus at drought onset compared with aCO_2_.

### Root exudation metabolites under drought and their potential implications

We found that under both CO_2_ levels, drought-stressed roots indeed exuded more secondary metabolites such as phenolics and terpenoids, in line with commonly reported upregulation of stress/defense pathways in plants under drought (Yadav et al. 2021, Bhatla and Lal 2023), confirming our second hypothesis. Although only a few metabolites mapped to each pathway, enrichment analysis of metabolites that increased under drought indicated drought-related shifts in glycine/serine/threonine metabolism, the one-carbon pool by folate and β-alanine metabolism (Table S4 available as Supplementary Data at *Tree Physiology* Online). These patterns collectively pointed to photorespiration-related and one-carbon/CoA-associated stress metabolism (Jabrin et al. 2003, Parthasarathy et al. 2019, Zhou et al. 2022, Rosa-Téllez et al. 2023). These changes may reflect both shoot-derived transport and local root processes supporting cellular maintenance and drought acclimation rather than growth-oriented allocation. In addition, the increase in secondary metabolites suggests that drought altered exudate composition in a selective manner, as these compounds are generally considered to be released via more tightly regulated, transporter-mediated processes, whereas many primary metabolites are more strongly influenced by concentration gradients and facilitated diffusion (Canarini et al. 2019). Further evidence for regulated allocation comes from the stronger representation of N-containing compounds among the drought-responsive metabolites. Given that trees grew in N-poor soil and in line with a surplus-C framework, we expected only a minor fraction of drought-responsive metabolites to be nitrogenous (Prescott et al. 2020). Instead, almost 50% of metabolites that increased under drought contained N. Consistently, the C:N ratio of the drought-responsive metabolites (not the absolute C:N ratio) was slightly lower than that of all metabolites included in the analysis (8.8 vs. 10.2). Under a strict surplus-C framework, this could imply relative N excess, contrary to the potentially N-limited conditions (Table S2 available as Supplementary Data at *Tree Physiology* Online; Prescott et al. 2020, Bunn et al. 2024). Together, this points to a regulated investment into root exudates rather than purely surplus-driven release.

Beyond their role in intracellular stress metabolism, several drought-enriched metabolites suggest roles in stress tolerance and potential rhizosphere interactions. Phenolic acids may contribute not only to oxidative stress protection but also to antimicrobial and signaling functions in the rhizosphere (Mandal et al. 2010, El-Nagar et al. 2020, Yadav et al. 2021, Saini et al. 2024). For example, caffeic acid has been linked to defence against *Ralstonia solanacearum*, and O-methylgallic acid has shown antimicrobial activity (Abhishek et al. 2019, Li et al. 2021). Amino acids, which showed the strongest increase under drought, may stimulate rhizosphere microorganisms and thereby indirectly enhance nutrient mobilization and N acquisition (Meier et al. 2017, Canarini et al. 2019). In line with this, β-alanine has been reported to promote *Bacillus* spore germination and facilitate root colonization (Tao et al. 2025). We also detected several osmoprotective compounds, including betaine, stachydrine, proline and trehalose, which are associated with drought tolerance in plants and, in some cases, in associated rhizobacteria (Sofy et al. 2020, He et al. 2024, Bhattacharyya et al. 2025). In addition, L-DOPA increased under drought, consistent with a possible role in plant defense signaling (Cascone et al. 2023). Overall, these results suggest that drought-induced shift in metabolites was not simply surplus-C release but a regulated and reversible shift toward stress-associated, often N-containing metabolites with potential roles in cellular protection and rhizosphere interactions.

### What drives root exudation?

Our findings support a dual-control framework in which transient surplus-C dynamics may have influenced exudation magnitude, whereas drought-induced regulation shaped exudate composition. In this view, C surplus theory and active C investment are not mutually exclusive, and higher C supply due to increased surplus C could determine how much C is available to release, while drought conditions and nutrient needs can influence where that C is allocated and which compounds are exuded.

Under eCO_2_ alone, photosynthesis increased, but growth kept pace, possibly leaving little surplus to send belowground, consistent with the surplus-C hypothesis, which predicts that when sinks can use available assimilates, exudation does not rise (Prescott et al. 2020, Bunn et al. 2024). Under drought, soluble sugars and exudation rose, particularly soluble sugars under eCO_2_, suggesting that growth restriction at drought onset may have temporarily increased belowground C availability. Typically, surplus C occurs under mild to moderate water deficits (Muller et al. 2011, Prescott 2022); the rapid decline in assimilation within 2 weeks under our severe drought treatment suggests that any surplus-C was transient, likely limited to early drought onset and potentially more pronounced under eCO_2_, where it may have contributed to the observed slight increase in exudation. Hence, our results do not support a sustained surplus-C state throughout the drought phase. Moreover, the shift toward secondary and N-containing metabolites under N-poor conditions is difficult to reconcile with a strict surplus-C disposal model. We therefore conclude that transient surplus C may have contributed to the amount of soluble sugar available, especially early in drought, but that the composition of exudates reflects an additional layer of selective allocation under stress.

## Conclusions

Our controlled experiment with pine saplings joins an increasing number of studies indicating a strong signal of increased root exudation under drought. To this, we add important information, facilitated by a unique combination of analyses:

(i) Drought-induced exudation was enriched in phenolic acids and amino acids including specific metabolites linked to stress metabolism and compounds with known roles in plant–microbe interactions.
(ii) Increased exudation under drought coincided with higher soluble sugar concentrations, particularly under combined drought and eCO_2_, and with lower starch reserves.

These observations should help improve our understanding of the roles of root exudation in the rhizosphere, particularly under future climate scenarios of increased drought and atmospheric CO_2_.

## Supplementary Material

Supplementary_8_tpag116

## Data Availability

The data supporting this study are available in Zenodo at https://doi.org/10.5281/zenodo.22710755.
