# Supplementary material for: Increased pine root exudation of phenolic acids and amino acids under drought
Source: Tree Physiol. 2026 Aug 20;46(9):tpag116. doi: 10.1093/treephys/tpag116 (PMC13601639; doi:10.1093/treephys/tpag116)
Supplement: Supplementary file 1 [file supplementary_8_tpag116.docx]

Supplementary Methods

Percent exudation per assimilation at whole sapling scale

Assimilation and root exudation fluxes were transformed into g C day^-1^, using the mass balance approach described in Klein and Hoch (2015). Briefly, total leaf area per sapling was calculated from leaf biomass, supplemented by biomass/ area ratio from Dror and Klein (2021). Assimilation flux was calculated for 10-h periods (time of light hours). Drought stressed trees were respiring, thus assimilation values were negative. Hence for visualization purposes only we registered 0.05 g C day^-1^ for drought stressed trees. Fine roots (<2 mm) were separated from the root system to determine their proportion of the total root system (n = 2 per treatment), with the resulting percentage (46.32 ± 5.99%) used as an estimate for C flux upscaling across treatments. Root exudation rate per root dry weight was summed along 24 h periods and across fine roots biomass. To account for biomass changes across time points, we estimated pre-drought and drought leaf and root biomass from post-drought values by scaling with stem diameter assuming linear allometry. For the drought time point, we used a linearly interpolated diameter between pre-drought and post-drought measurements to minimize bias from transient drought-related stem shrinkage.

Non-structural carbohydrate analysis

Soluble sugar and starch extractions were conducted following the standard and widely validated methods of Landhäusser et al. (2018). Approximately 30 ± 1 mg of each dried sample was extracted in 80% ethanol at 90 °C for 15 min. For fine roots, a smaller sample mass was used (≥15 mg) due to limited material. The supernatant was used for soluble sugar analysis. Ethanol was evaporated (60 °C) and the residue was reconstituted with 1 mL of HPLC-grade water. Simultaneously, the remaining pellet, containing starch, was washed three times with ethanol before undergoing enzymatic hydrolysis (see below). Soluble sugars, glucose and fructose, were analyzed using an ultra-fast liquid chromatography (UFLC) system (Prominence UFLC, Shimadzu Scientific Instruments, Kyoto, Japan) equipped with a SIL-20AC autosampler, CTO-20AC column oven, and RID-20A detector. Chromatographic separation was achieved using an Aminex HPX-87C column (300 × 7.8 mm, 9 μm particle size) paired with a micro-guard IG carbo C guard cartridge (Bio-Rad, CA, USA). The column temperature was maintained at 84°C, with water as the mobile phase at a flow rate of 0.6 mL/min, following the manufacturer’s guidelines. Retention times for sugars were established using a carbohydrate standard mix (Bio-Rad, CA, USA). Quantification of glucose and fructose was based on calibration curves generated from standard solutions (Sigma, catalog numbers 47829 and F2793, respectively).

Starch hydrolysis was performed using α-amylase (*Bacillus licheniformis*, Sigma A4551) for 120 min at 85 °C, followed by amyloglucosidase (*Aspergillus niger*, Roche #11202367001, ROAMYGLL) for 30 min at 55 °C. Glucose in the resulting digest was then quantified using the Glucose Assay Reagent (Cat. No. G3293-50ML, Sigma-Aldrich, St. Louis, MO, USA), based on a coupled hexokinase/glucose-6-phosphate dehydrogenase assay, and absorbance was read at 340 nm with a microplate reader (Infinite M200 Pro; Tecan, Männedorf, Switzerland). Dry matter percentages of soluble sugars and starch were calculated in accordance with the standardized protocols of Landhäusser et al. (2018) and expressed as % (w/w) relative to dry matter.

Metabolite analysis of root exudates using LC-MS
Following freeze-drying, samples for metabolite analysis were resuspended in 1 mL methanol (1:1, v), vortexed, and centrifuged (4 °C, 2 min, 1,000 × rcf). The resulting supernatant was divided for polar and semi-polar metabolite profiling, dried under nitrogen gas, and reconstituted in solvents specific to each analytical method. A pooled QC sample was generated by combining 60 µL from each sample.

*Analysis of polar metabolites*

For polar metabolite profiling, extracts were resuspended in 150 µL acetonitrile (1:1, vol/vol) and supplemented with isotope-labeled standards prior to injection. Ultra-high-performance liquid chromatography coupled to high-resolution mass spectrometry was carried out on a Vanquish system connected to a Q Exactive HF Hybrid Quadrupole-Orbitrap mass spectrometer (Thermo Fisher Scientific) equipped with electrospray ionization (ESI). Separation was achieved by hydrophilic interaction liquid chromatography (HILIC) using an ACQUITY Premier BEH Amide column (1.7 μm, 2.1 mm × 100 mm; Waters). The chromatographic program consisted of a two-step gradient lasting 12 min, followed by 4 min re-equilibration, at a flow rate of 450 μL min⁻¹, column temperature of 30 °C, and injection volume of 2 μL. Mobile phase A contained 10 mM ammonium formate in acetonitrile (pH 10), and mobile phase B contained 10 mM ammonium formate in 40% acetonitrile (pH 10) with 0.1% medronic acid. Mass spectra were acquired in both positive and negative ionization modes at 120,000 resolution over a scan range of 60–900 m/z, using iterative data-dependent MS/MS acquisition (TopN = 10; NCE = 20, 40, 60). The pooled QC sample was analyzed in MS/MS mode to support compound identification.

*Analysis of semi-polar metabolites*

For semi-polar metabolite analysis, samples were resuspended in 120 µL water:methanol acid (89.9:10.0:0.1, vol/vol/vol, containing 10 mM ammonium formate), diluted fivefold in mobile phase A, and spiked with isotope-labeled standards. Mobile phase A consisted of 10 mM ammonium formate and 0.1% formic acid in water, whereas mobile phase B consisted of 10 mM ammonium formate in methanol with 0.1% formic acid. Chromatographic separation was performed on an ACQUITY UPLC HSS T3 C18 column (2.1 × 150 mm, 1.8 μm; Waters). Semi-polar metabolites were analyzed using a modified version of the protocol described by Doneanu et al. (2011), coupled to the same HRMS setup used for the polar metabolite analysis.

For both polar and semi-polar datasets, peak alignment and area extraction were performed using Compound Discoverer 3.3 (Thermo Scientific, MA, USA) and Skyline 23.1 (Adams et al., 2020). Chromatographic alignment was conducted with an adaptive curve setting (maximum 0.4 min, 4 ppm), after which peaks were grouped across injections based on retention time and accurate mass. Metabolites were annotated at four confidence levels: Level 1, based on retention time matched to authentic in-house standards, accurate mass (±3 ppm), and MS/MS spectra; Level 2a, based on retention time and accurate mass (±3 ppm) matched to authentic in-house standards; Level 2b, based on accurate mass and MS/MS spectra matched to external libraries; and Level 3, based on accurate mass only matched to external libraries. Only Level 1 and Level 2a annotations were retained for downstream analyses. Peak areas were normalized to root dry weight (Williams et al., 2022) to compare treatment effects on the quantity of each metabolite exuded per unit biomass and thereby estimate specific metabolite C fluxes rather than relative composition alone. In contrast, normalization to total C per sample would only reflect the proportion of each metabolite within the complete exudate mixture and would not capture treatment-related differences in metabolite export from roots. Fifty-seven metabolites were removed from further analysis because their abundances were lower than those of blanks in more than 33% of the sample groups across the measured time points. Carbon-to-nitrogen (C) ratios were calculated from the molecular formula of each identified metabolite by summing the total numbers of C and N atoms.

Supplementary Tables

**Table S1**. Number of replicates for each measurement across time points and treatments (C = aCO2-WW, D = aCO2-DS, E = eCO_2_-WW, E×D = eCO2-DS; DS = drought stressed, WW = well watered)

|  | **Pre-drought** | | | | **Drought** | | | | **Post-drought** | | | |
| --- | --- | --- | --- | --- | --- | --- | --- | --- | --- | --- | --- | --- |
|  | **C** | **D** | **E** | **E×D** | **C** | **D** | **E** | **E×D** | **C** | **D** | **E** | **E×D** |
| assimilation | 8 | 8 | 8 | 8 | 7 | 8 | 7 | 7 | 7 | 6 | 8 | 7 |
| Soluble sugar - branchlet | 8 | 8 | 8 | 8 | 7 | 8 | 8 | 7 | 8 | 8 | 8 | 8 |
| Soluble sugar - fine roots | 8 | 8 | 8 | 8 | 7 | 8 | 7 | 6 | 8 | 8 | 8 | 8 |
| Starch - branchlet | 8 | 8 | 8 | 8 | 8 | 8 | 8 | 8 | 8 | 8 | 8 | 8 |
| Starch - fine roots | 7 | 7 | 7 | 8 | 8 | 8 | 7 | 7 | 8 | 8 | 8 | 7 |
| Root exudation | 8 | 8 | 8 | 8 | 8 | 8 | 8 | 8 | 8 | 8 | 8 | 8 |
| Stem water potential | 8 | 8 | 8 | 8 | 7 | 8 | 8 | 7 | 8 | 8 | 8 | 8 |
| branch biomass |  |  |  |  |  |  |  |  | 8 | 7 | 8 | 8 |
| needle biomass |  |  |  |  |  |  |  |  | 8 | 7 | 8 | 8 |
| stem biomass |  |  |  |  |  |  |  |  | 8 | 8 | 8 | 8 |
| root biomass |  |  |  |  |  |  |  |  | 8 | 8 | 8 | 8 |
| Metabolites of root exudation | 5 | 5 | 5 | 5 | 5 | 5 | 5 | 5 | 5 | 5 | 5 | 5 |

**Table S2**. Results for pH, EC, nitrate and soil organic C (SOC) of the soil for single and combined CO_2_ and drought treatment (DS = drought stressed, WW = well watered) at Drought and Post-drought (n=6).

| **Time** | **Treatment group** | **pH** | **EC (ds m^-1^)** | **NO_3_ (mg kg^-1^)** | **SOC (g C kg^-1^)** |
| --- | --- | --- | --- | --- | --- |
| Drought | aCO2-WW | 7.9±0.1 | 432.6±155.7 | 0.5±0.3 | 18.1±4.9 |
|  | aCO2-DS | 8.0±0.1 | 337.8± 62.0 | 0.6±0.3 | 15.9±1.2 |
|  | eCO2-WW | 7.7±0.2 | 440.4±137.5 | 0.6±0.4 | 16.4±2.6 |
|  | eCO2-DS | 7.6±0.2 | 497.7±169.5 | 0.2±0.3 | 16.7±1.6 |
| Post-drought | aCO2-WW | 7.8±0.1 | 353.5±113.4 | 0.9±0.6 | 16.9±1.0 |
|  | aCO2-DS | 7.9±0.1 | 261.4± 52.4 | 1±0.5 | 17.4±1.4 |
|  | eCO2-WW | 7.7±0.2 | 305.2± 76.2 | 0.2±0.1 | 16.8±3.3 |
|  | eCO2-DS | 7.7±0.2 | 316.9± 80.8 | 0.5±0.5 | 17.6±1.4 |

**Table S3**. Change in root exudation rate between flushes (Δ = Flush 2 − Flush 1 in μg C cm⁻² day⁻¹) for single and combined CO_2_ and drought treatment (DS = drought stressed, WW = well watered) at Pre-drought, Drought and Post-Drought (n=8). Positive values indicate an increase from the first to the second flush, whereas negative values indicate a decrease.

| **Time** | **Treatment** | **Δ exudation rate (± SD)** |
| --- | --- | --- |
| Pre-drought | aCO2-WW | 2.14 ± 0.65 |
|  | aCO2-DS | 1.54 ± 2.49 |
|  | eCO2-WW | 1.50 ± 0.95 |
|  | eCO2-DS | 2.13 ± 1.40 |
| Drought | aCO2-WW | 0.98 ± 1.30 |
|  | aCO2-DS | -2.45 ± 2.29 |
|  | eCO2-WW | 1.62 ± 0.65 |
|  | eCO2-DS | -2.98 ± 3.91 |
| Post-drought | aCO2-WW | 1.71 ± 1.15 |
|  | aCO2-DS | 0.94 ± 1.44 |
|  | eCO2-WW | 1.06 ± 0.59 |
|  | eCO2-DS | 0.94 ± 1.17 |

**Table S4**. Metabolic pathways that were identified based on root exudate metabolites that increased significantly in aCO_2_-DS and/or eCO_2_ -DS at the drought time point (total of 55 metabolites; DS = drought stressed). Only pathways with p ≤ 0.1 are listed. Hits represent the number of metabolites identified in the dataset, as part of all metabolites in a given pathway.

| **Pathways** | **hits** | **p-value** |
| --- | --- | --- |
| Glycine, serine and threonine metabolism | 3/33 | 0.012 |
| beta-Alanine metabolism | 2/18 | 0.029 |
| One carbon pool by folate | 2/21 | 0.039 |
| Betalain biosynthesis | 1/3 | 0.045 |
| Pantothenate and CoA biosynthesis | 2/25 | 0.053 |
| Cyanoamino acid metabolism | 2/29 | 0.070 |
| Glyoxylate and dicarboxylate metabolism | 2/29 | 0.070 |
| Isoquinoline alkaloid biosynthesis | 1/6 | 0.088 |
| Purine metabolism | 3/75 | 0.101 |

**Table S5**. Results for linear (mixed) models of drought treatment, CO_2_ treatment and time points of measurements (Time) and their interaction effects. Linear mixed models (LMM) were used for all variables except for the variables needle, branch, stem and root, where linear models were used. Exudation was modeled using a GLMM with a Tweedie distribution and chi-square (χ^2^) statistics are reported. Individual trees were included as random effect in the linear mixed models. Exudation Significant differences are in boldface.

**Table S6**. Post hoc analyses derived from the LMMs, including a Tweedie GLMM for root exudation. Tests show phase-specific treatment effects, CO₂-specific drought × time effects and drought–control comparisons within each CO₂ level. P-values were Holm-adjusted, significant differences are in boldface; note d indicates reduced df₁ due to linear dependence.

| **Variable** | **Follow-up analysis** | **Effect / contrast** | **CO2** | **Phase / phase comparison** | **df1** | **df2 / df** | **F / χ^2^/ t / z ratio** | **Holm-adjusted p** |
| --- | --- | --- | --- | --- | --- | --- | --- | --- |
| Net Photosynthesis | Phase-specific joint test | co2 |  | Pre-drought | 1 | 70.9 | 55.17 | **<0.001** |
|  | Phase-specific joint test | co2:drought |  | Pre-drought | 1 | 70.9 | 0.30 | 1.000 |
|  | Phase-specific joint test | drought |  | Pre-drought | 1 | 70.9 | 0.00 | 1.000 |
|  | Phase-specific joint test | co2 |  | Drought | 1 | 72.7 | 0.53 | 0.471 |
|  | Phase-specific joint test | co2:drought |  | Drought | 1 | 72.7 | 14.64 | **<0.001** |
|  | Phase-specific joint test | drought |  | Drought | 1 | 72.7 | 461.77 | **<0.001** |
|  | Phase-specific joint test | co2 |  | Post-drought | 1 | 73.4 | 43.95 | **<0.001** |
|  | Phase-specific joint test | co2:drought |  | Post-drought | 1 | 73.4 | 0.42 | 1.000 |
|  | Phase-specific joint test | drought |  | Post-drought | 1 | 73.4 | 0.32 | 1.000 |
|  | Drought x time omnibus within CO2 | drought:t (note d) | aCO2 |  | 2 | 51.5 | 61.17 | **<0.001** |
|  | Drought x time omnibus within CO2 | drought:t (note d) | eCO2 |  | 2 | 52.3 | 138.00 | **<0.001** |
|  | Drought vs control within phase | drought - control | aCO2 | Pre-drought |  | 70.9 | -0.37 | 0.816 |
|  | Drought vs control within phase | drought - control | eCO2 | Pre-drought |  | 70.9 | 0.40 | 1.000 |
|  | Drought vs control within phase | drought - control | aCO2 | Drought |  | 72.2 | -12.68 | **<0.001** |
|  | Drought vs control within phase | drought - control | eCO2 | Drought |  | 73.2 | -17.64 | **<0.001** |
|  | Drought vs control within phase | drought - control | aCO2 | Post-drought |  | 74.3 | -0.83 | 0.816 |
|  | Drought vs control within phase | drought - control | eCO2 | Post-drought |  | 72.2 | 0.06 | 1.000 |
| Soluble sugar branchlet | Phase-specific joint test | co2 |  | Pre-drought | 1 | 80.7 | 3.42 | 0.068 |
|  | Phase-specific joint test | co2:drought |  | Pre-drought | 1 | 80.7 | 0.84 | 0.363 |
|  | Phase-specific joint test | drought |  | Pre-drought | 1 | 80.7 | 3.96 | 0.050 |
|  | Phase-specific joint test | co2 |  | Drought | 1 | 81.0 | 19.16 | **<0.001** |
|  | Phase-specific joint test | co2:drought |  | Drought | 1 | 81.0 | 27.06 | **<0.001** |
|  | Phase-specific joint test | drought |  | Drought | 1 | 81.0 | 21.79 | **<0.001** |
|  | Phase-specific joint test | co2 |  | Post-drought | 1 | 80.7 | 10.45 | **0.004** |
|  | Phase-specific joint test | co2:drought |  | Post-drought | 1 | 80.7 | 3.27 | 0.149 |
|  | Phase-specific joint test | drought |  | Post-drought | 1 | 80.7 | 10.09 | **0.004** |
|  | Drought x time omnibus within CO2 | drought:t (note d) | aCO2 |  | 2 | 53.8 | 0.56 | 0.574 |
|  | Drought x time omnibus within CO2 | drought:t (note d) | eCO2 |  | 2 | 53.8 | 7.44 | **0.003** |
|  | Drought vs control within phase | drought - control | aCO2 | Pre-drought |  | 80.6 | 0.76 | 1.000 |
|  | Drought vs control within phase | drought - control | eCO2 | Pre-drought |  | 80.6 | 2.05 | **0.043** |
|  | Drought vs control within phase | drought - control | aCO2 | Drought |  | 81.0 | -0.38 | 1.000 |
|  | Drought vs control within phase | drought - control | eCO2 | Drought |  | 81.0 | 6.98 | **<0.001** |
|  | Drought vs control within phase | drought - control | aCO2 | Post-drought |  | 80.6 | 0.97 | 1.000 |
|  | Drought vs control within phase | drought - control | eCO2 | Post-drought |  | 80.6 | 3.52 | **<0.001** |
| Soluble sugar root | Phase-specific joint test | co2 |  | Pre-drought | 1 | 75.0 | 9.42 | **0.009** |
|  | Phase-specific joint test | co2:drought |  | Pre-drought | 1 | 75.0 | 0.36 | 1.000 |
|  | Phase-specific joint test | drought |  | Pre-drought | 1 | 75.0 | 4.04 | 0.096 |
|  | Phase-specific joint test | co2 |  | Drought | 1 | 77.1 | 5.28 | 0.049 |
|  | Phase-specific joint test | co2:drought |  | Drought | 1 | 77.1 | 9.97 | **0.007** |
|  | Phase-specific joint test | drought |  | Drought | 1 | 77.1 | 32.78 | **<0.001** |
|  | Phase-specific joint test | co2 |  | Post-drought | 1 | 75.0 | 0.01 | 0.936 |
|  | Phase-specific joint test | co2:drought |  | Post-drought | 1 | 75.0 | 0.00 | 1.000 |
|  | Phase-specific joint test | drought |  | Post-drought | 1 | 75.0 | 0.22 | 0.643 |
|  | Drought x time omnibus within CO2 | drought:t (note d) | aCO2 |  | 2 | 53.0 | 2.05 | 0.140 |
|  | Drought x time omnibus within CO2 | drought:t (note d) | eCO2 |  | 2 | 53.8 | 14.67 | **<0.001** |
|  | Drought vs control within phase | drought - control | aCO2 | Pre-drought |  | 75.0 | 1.85 | 0.191 |
|  | Drought vs control within phase | drought - control | eCO2 | Pre-drought |  | 75.0 | 1.00 | 0.643 |
|  | Drought vs control within phase | drought - control | aCO2 | Drought |  | 76.1 | 1.88 | 0.191 |
|  | Drought vs control within phase | drought - control | eCO2 | Drought |  | 77.8 | 6.08 | **<0.001** |
|  | Drought vs control within phase | drought - control | aCO2 | Post-drought |  | 75.0 | -0.35 | 0.728 |
|  | Drought vs control within phase | drought - control | eCO2 | Post-drought |  | 75.0 | -0.31 | 0.758 |
| Starch branchlet | Phase-specific joint test | drought |  | Pre-drought | 1 | 83.2 | 0.72 | 0.400 |
|  | Phase-specific joint test | drought |  | Drought | 1 | 83.2 | 57.59 | **<0.001** |
|  | Phase-specific joint test | drought |  | Post-drought | 1 | 83.2 | 24.86 | **<0.001** |
|  | Drought x time omnibus within CO2 | drought:t (note d) | aCO2 |  | 2 | 56.0 | 17.50 | **<0.001** |
|  | Drought x time omnibus within CO2 | drought:t (note d) | eCO2 |  | 2 | 56.0 | 28.16 | **<0.001** |
|  | Drought vs control within phase | drought - control | aCO2 | Pre-drought |  | 83.2 | 0.81 | 0.421 |
|  | Drought vs control within phase | drought - control | eCO2 | Pre-drought |  | 83.2 | 0.39 | 0.700 |
|  | Drought vs control within phase | drought - control | aCO2 | Drought |  | 83.2 | -5.42 | **<0.001** |
|  | Drought vs control within phase | drought - control | eCO2 | Drought |  | 83.2 | -5.32 | **<0.001** |
|  | Drought vs control within phase | drought - control | aCO2 | Post-drought |  | 83.2 | 2.15 | 0.069 |
|  | Drought vs control within phase | drought - control | eCO2 | Post-drought |  | 83.2 | 4.90 | **<0.001** |
| Starch root | Phase-specific joint test | co2 |  | Pre-drought | 1 | 78.0 | 10.68 | **0.005** |
|  | Phase-specific joint test | co2:drought |  | Pre-drought | 1 | 78.0 | 0.96 | 0.349 |
|  | Phase-specific joint test | drought |  | Pre-drought | 1 | 78.0 | 0.01 | 1.000 |
|  | Phase-specific joint test | co2 |  | Drought | 1 | 78.0 | 8.92 | **0.008** |
|  | Phase-specific joint test | co2:drought |  | Drought | 1 | 78.0 | 2.08 | 0.349 |
|  | Phase-specific joint test | drought |  | Drought | 1 | 78.0 | 19.36 | **<0.001** |
|  | Phase-specific joint test | co2 |  | Post-drought | 1 | 78.0 | 5.70 | **0.019** |
|  | Phase-specific joint test | co2:drought |  | Post-drought | 1 | 78.0 | 2.52 | 0.349 |
|  | Phase-specific joint test | drought |  | Post-drought | 1 | 78.0 | 0.30 | 1.000 |
|  | Drought x time omnibus within CO2 | drought:t (note d) | aCO2 |  | 2 | 52.9 | 3.77 | 0.059 |
|  | Drought x time omnibus within CO2 | drought:t (note d) | eCO2 |  | 2 | 54.9 | 3.71 | 0.059 |
|  | Drought vs control within phase | drought - control | aCO2 | Pre-drought |  | 78.0 | 0.61 | 0.542 |
|  | Drought vs control within phase | drought - control | eCO2 | Pre-drought |  | 78.0 | -0.77 | 0.884 |
|  | Drought vs control within phase | drought - control | aCO2 | Drought |  | 78.0 | -2.17 | 0.100 |
|  | Drought vs control within phase | drought - control | eCO2 | Drought |  | 78.0 | -4.00 | **<0.001** |
|  | Drought vs control within phase | drought - control | aCO2 | Post-drought |  | 78.0 | 1.54 | 0.257 |
|  | Drought vs control within phase | drought - control | eCO2 | Post-drought |  | 78.0 | -0.72 | 0.884 |
| Diameter | Phase-specific joint test | co2 |  | Pre-drought | 1 | 31.5 | 9.01 | **0.005** |
|  | Phase-specific joint test | drought |  | Pre-drought | 1 | 31.5 | 0.04 | 1.000 |
|  | Phase-specific joint test | co2 |  | Drought | 1 | 31.5 | 21.28 | **<0.001** |
|  | Phase-specific joint test | drought |  | Drought | 1 | 31.5 | 10.24 | 0.009 |
|  | Phase-specific joint test | co2 |  | Post-drought | 1 | 31.5 | 21.87 | **<0.001** |
|  | Phase-specific joint test | drought |  | Post-drought | 1 | 31.5 | 0.22 | 1.000 |
|  | Drought x time omnibus within CO2 | drought:t (note d) | aCO2 |  | 2 | 56.0 | 13.73 | **<0.001** |
|  | Drought x time omnibus within CO2 | drought:t (note d) | eCO2 |  | 2 | 56.0 | 24.74 | **<0.001** |
|  | Drought vs control within phase | drought - control | aCO2 | Pre-drought |  | 31.5 | 0.80 | 0.857 |
|  | Drought vs control within phase | drought - control | eCO2 | Pre-drought |  | 31.5 | -0.53 | 0.644 |
|  | Drought vs control within phase | drought - control | aCO2 | Drought |  | 31.5 | -1.27 | 0.645 |
|  | Drought vs control within phase | drought - control | eCO2 | Drought |  | 31.5 | -3.26 | **0.008** |
|  | Drought vs control within phase | drought - control | aCO2 | Post-drought |  | 31.5 | 0.35 | 0.857 |
|  | Drought vs control within phase | drought - control | eCO2 | Post-drought |  | 31.5 | -1.01 | 0.644 |
| Exudation | Phase-specific joint test | drought |  | Pre-drought | 1 | Inf | 4.97 | 0.051 |
|  | Phase-specific joint test | drought |  | Drought | 1 | Inf | 54.24 | **<0.001** |
|  | Phase-specific joint test | drought |  | Post-drought | 1 | Inf | 0.13 | 0.715 |
|  | Drought x time omnibus within CO2 | drought:t (note d) | aCO2 |  | 2 | Inf | 5.96 | 0.051 |
|  | Drought x time omnibus within CO2 | drought:t (note d) | eCO2 |  | 2 | Inf | 17.56 | **<0.001** |
|  | Drought vs control within phase | drought / control | aCO2 | Pre-drought |  | Inf | 1.27 | 0.405 |
|  | Drought vs control within phase | drought / control | eCO2 | Pre-drought |  | Inf | 1.91 | 0.113 |
|  | Drought vs control within phase | drought / control | aCO2 | Drought |  | Inf | 4.09 | **<0.001** |
|  | Drought vs control within phase | drought / control | eCO2 | Drought |  | Inf | 6.28 | **<0.001** |
|  | Drought vs control within phase | drought / control | aCO2 | Post-drought |  | Inf | 0.38 | 0.702 |
|  | Drought vs control within phase | drought / control | eCO2 | Post-drought |  | Inf | 0.13 | 0.893 |

**Table S7**. Results for Permanova for PCA (Fig. 4B) of Metabolites for Drought treatment, CO_2_ treatment and time points of measurements (Time) and their interaction effects. Significant differences are in boldface.

|  | **Df** | **SumOfSqs** | **R^2^** | **F** | **p-value** |
| --- | --- | --- | --- | --- | --- |
| Drought | 1 | 0.014 | 0.058 | 5.509 | 0.008 |
| CO_2_ | 1 | 0.003 | 0.014 | 1.333 | 0.231 |
| Time | 2 | 0.055 | 0.232 | 11.037 | 0.001 |
| Drought*CO_2_ | 1 | 0.006 | 0.025 | 2.338 | 0.077 |
| Drought*Time | 2 | 0.030 | 0.126 | 5.989 | 0.001 |
| CO_2_*Time | 2 | 0.007 | 0.031 | 1.465 | 0.199 |
| Drought*CO_2_*Time | 2 | 0.002 | 0.009 | 0.426 | 0.885 |
| Residual | 48 | 0.120 | 0.505 | NA | NA |
| Total | 59 | 0.238 | 1.000 | NA | NA |

**Table S8**. Results for Permanova for PCA (Fig. 6B), for Drought treatment, CO_2_ treatment and time points of measurements (Time) and their interaction effects on all measured variables: biomass (needle, stem, branch and root), assimilation, soluble sugar, starch and root exudates; Significant differences are in boldface.

|  | **Df** | **SumOfSqs** | **R^2^** | **F** | **p-value** |
| --- | --- | --- | --- | --- | --- |
| Drought | 1 | 3.70E-02 | 0.046 | 7.315 | **0.006** |
| CO_2_ | 1 | 3.06E-01 | 0.381 | 60.420 | **0.001** |
| Time | 2 | 1.39E-02 | 0.017 | 1.377 | 0.252 |
| Drought*CO_2_ | 1 | 1.14E-02 | 0.014 | 2.252 | 0.118 |
| Drought*Time | 2 | 1.30E-02 | 0.016 | 1.283 | 0.297 |
| CO_2_*Time | 2 | -1.71E-03 | -0.002 | -0.169 | 1 |
| Drought*CO_2_*Time | 2 | -2.94E-03 | -0.004 | -0.291 | 1 |
| Residual | 84 | 4.25E-01 | 0.530 | NA | NA |
| Total | 95 | 8.02E-01 | 1.000 | NA | NA |

Supplementary Figures

**Figure S1.** Experimental conditions and setup. Rhizobox set up used in the experiment (A) and CO_2_ concentration recorded at 15-min intervals over the experimental period, black line marks 850 ppm and DRD = 0 is added (Days relative to drought = DRD; A). Rhizobox set up used in the experiment.

**Figure S2.** Stem water potential of pines at pre-drought, drought (grey background) and post-drought under single and combined CO_2_ and drought treatment (DS = drought stressed, WW = well watered). Lines in boxes represent the median; the top and bottom of boxes represent first and third quartiles and whiskers represent the 1.5 interquartile range.

**Figure S3.** Net photosynthesis rate (A), stomatal conductance (B) and intrinsic water-use efficiency (iWUE; C) measured over the period of the experiment, single and combined CO_2_ and drought treatment (DS = drought stressed, WW = well watered). Grey background indicates the drought period. Means are depicted with standard error bars (n = 1-8).

**Figure S4.** Metabolic profiles of root exudates of pines at pre-drought, drought and post-drought presented in a heatmap. Values of heatmap are shown as original log_2_-transformed peak areas for each sample under single and combined CO_2_ and drought treatment (DS = drought stressed, WW = well watered). Only metabolites with a fold-change (FC) > 1 or < -1 that changed significantly are presented (FC of each treatment group compared to the control group).

**Figure S5.** Cohen's d effect sizes for pairwise comparisons of each treatment group compared to the control group for pre-drought, drought and post-drought (A) on complete cases only (without imputation). Error bars represent 95% confidence intervals of the effect sizes; asterisks significant difference of the comparison. PCA at pre-drought, drought and post-drought under single and combined CO_2_ and drought treatment including all variables shown in A on complete cases only (without imputation; DS = drought stressed, WW = well watered).

**Figure S6.** Height (cm) of pines at pre-drought, drought and post-drought under single and combined CO_2_ and drought treatment (DS = drought stressed, WW = well watered). Means are depicted with standard error bars. Grey background indicates the drought period. No significant difference was found between the treatments.

**Figure S7.** Percent exudation of assimilation per tree (%; n = 5-8; A) and percent exudation (gC per day) per biomass (g; %; n = 5-8; B) of pines at pre-drought, drought (grey background) and post-drought under single and combined CO_2_ and drought treatment (DS = drought stressed, WW = well watered). Lines in boxes represent the median, the top and bottom of boxes represent first and third quartiles and whiskers represent the 1.5 interquartile range. Significant difference between treatments within one time point is indicated by letters based on Post Hoc Comparison with Bonferroni correction (p < 0.05). The y-axis is displayed on a pseudo logarithmic scale.
† For drought-stressed trees, percent exudation was calculated using an assimilation value of 0.05 gC day^-1^ for visualization purposes only, as these trees were respiring and thus had negative assimilation values.

**Figure S8.** Venn diagram of 57 metabolites of root exudates that changed significantly with FC >1 or < -1, at the time point drought (left panel) and post-drought (right panel; DS = drought stressed, WW = well watered). ^†^ One metabolite is overlapping between the time point drought and post-drought.

**Figure S9.** Sensitivity check of correlation of root exudation rate with percent soluble sugar and starch in fine roots (w/w), with 1 outlier taken out (A) and 2 outliers taken out (B) for single and combined eCO_2_ and drought treatment (DS = drought stressed, WW = well watered).

**Figure S10.** Continuous SWC (% v/v) of Rhizoboxes from drought onset until the end of the experiment under single and combined CO_2_ and drought treatment measured horizontally at 25 cm depth (n=1-2; A; DS = drought stressed, WW = well watered). SWC (% v/v) of rhizoboxes at pre-drought, drought and post-drought under single and combined CO_2_ and drought treatment measured vertically at 0-20 cm depth (B). Grey background indicates the drought period. Lines in boxes represent the median; the top and bottom of boxes represent first and third quartiles and whiskers represent the 1.5 interquartile range.
